# Supplementary material for: A systematic analysis of regression models for protein engineering
Source: PLoS Comput Biol. 2024 May 3;20(5):e1012061. doi: 10.1371/journal.pcbi.1012061 (PMC11095727; doi:10.1371/journal.pcbi.1012061)
Supplement: S1 File — Contains dataset overview, additional results, calibration definitions, protocol descriptions. (PDF) [file pcbi.1012061.s001.pdf]

SUPPORTING INFORMATION

A systematic analysis of regression models for protein engineering

Richard Michael[1¶], Jacob Kæstel-Hansen[2¶], Peter Mørch Groth[1,3], Simon Bartels[1], Jesper Salomon[3], Pengfei Tian[4], Nikos S. Hatzakis[2], Wouter Boomsma[1*],

1 Department of Computer Science, University of Copenhagen, Copenhagen, Denmark
2 Department of Chemistry, University of Copenhagen, Copenhagen, Denmark
3 Enzyme Research, Novonesis, Kongens Lyngby, Denmark
4 Enzyme Research, Novozymes A/S, Kongens Lyngby, Denmark

¶These authors contributed equally to this work.
* wb@di.ku.dk

1 DATA

For each protein we have experimental observations from deep mutational scans (DMS), which record growth under chemical stress - β-Lactamase, Ubiquitin, BRCA1, ParD-antitoxin, and thermal TIM-Barrel (see table A cf. supplementary material in [1]).

Table A. Overview of the Data.

DATASET	REF.	EFFECT	STRESS TYPE
β-Lactamase	[2]	growth	CHEMICAL
Ubiquitin	[3]	growth	CHEMICAL
Calmodulin	[4]	growth	CHEMICAL
TIM-Barrel	[5]	growth	THERMAL
BRCA1	[6]	growth	CHEMICAL
T2-MTH	[7]	growth	CHEMICAL/GENETIC
ParD-antitoxin	[8]	growth	CHEMICAL

2 COMPUTING INFRASTRUCTURE

All experiments were initially run on an Apple M1 Pro (using the TensorFlow Metal support), and subsequently a compute cluster with Intel Xeon 6248 CPUs, running a Linux kernel 4.18.0-425.3.1.e18.x86_64 and NVIDIA Quadro RTX 6000 (Driver Vers. 525.60.13), NVIDIA TITAN X, using CUDA 11.6 , 11.8, and 12.0 . Representations were obtained using the aforementioned compute cluster with respective specifications.

3 ADDITIONAL RESULTS

3.1 RANK CORRELATION

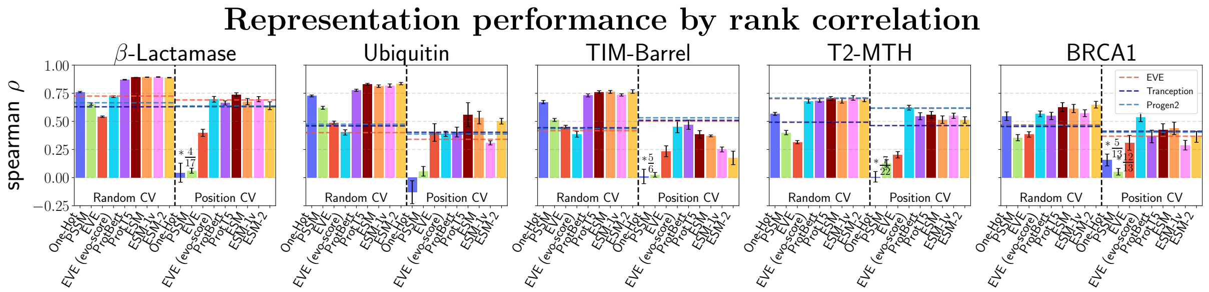

Fig A. Representation comparison of GP (Matérn$\frac{5}{2}$) by spearman rank-correlation. Dashed lines are unsupervised methods (no regression-head) - ensemble predictions of latent variable models EVE, Tranception (model M), [1, 9, 10] and Progen2, computed on the ProteinGym [10]; correlations of predicted likelihoods computed with the corresponding DMS experimental values. For unsupervised baselines also different protocols apply for which mean values are reported: *Random CV* is a standard 10-fold CV protocol, and *Position CV* includes specific mutation positions (in steps of 15) as assessment. We note that these protocols for the unsupervised comparison are not exactly the same as the supervised regression setting discussed in the Methods section, since pre-computed ProteinGym results were used and no buffer positions included. The fraction annotations indicate number of computable results i.e. if the predictions are constant as is the case for some data-splits with one-hot input the *spearman ρ* cannot be computed and only the results of splits are presented, which are not constant.

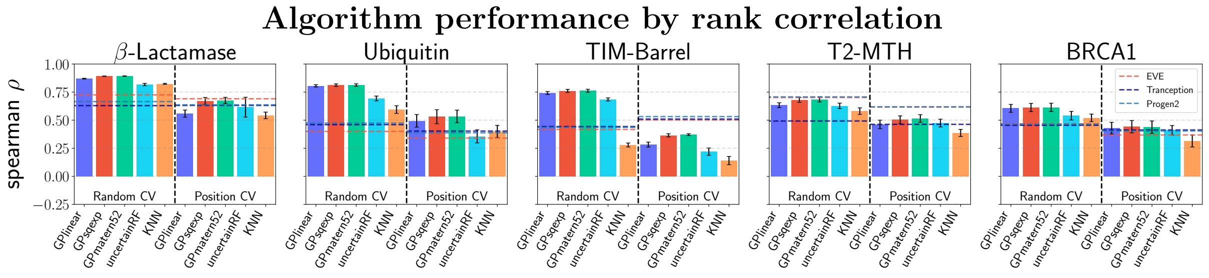

Fig B. Algorithm comparison on ESM-1b representation by rank-correlation. Dashed lines are unsupervised methods (no regression-head) - ensemble predictions of latent variable models EVE, Tranception (model M) [1, 9, 10] and Progen2, computed from the ProteinGym [10]: correlations of predicted likelihoods computed with the corresponding DMS experimental values. For unsupervised baselines also different protocols apply for which mean values are reported: *Random CV* is a standard 10-fold CV protocol, and *Position CV* includes specific mutation positions (in steps of 15) as assessment. We note that these protocols for the unsupervised comparison are not exactly the same as the supervised regression setting discussed in the Methods section, since pre-computed ProteinGym results were used and no buffer positions included.

3.2 REPRESENTATION UPDATES

We consider the development and different implementations of existing representation models such as from the *ESM*-family: ESM-1b, ESM-1v, and ESM-2; as well as the ProtBert related updated ProtT5. While the performance across *PLM* based models is consistently

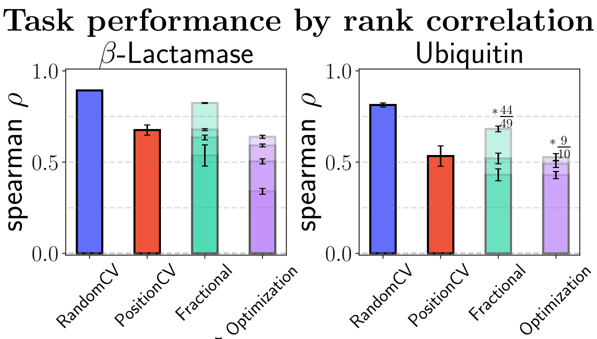

Fig C. Task comparison of GP (Matérn$\frac{5}{2}$ on ESM-1B) by rank-correlation. The annotations indicate the fraction of computable results.

high, we observe that ESM-2 appears to (generally) not be an improvement compared to ESM-1B or ESM-1V. Here the rank correlations is largely preserved, while accuracy decreases and the contrast between splitting strategies is pronounced (Figures A and D). In contrast there is some improvement when choosing ProtT5 over ProtBert, especially when it comes to positional extrapolation.

3.2.1 Deriving PSSMs from Sequence Alignments

The presented PSSM has been derived from *hmmer* HMMs [11, 12, 13] such that for each protein MSA (element of the *ProteinGym* [10]) an HMM has been built, and the transition matrices used to compute positional log-odds; we account for UniProtKB/TrEMBL backround statistics of the amino acids (time-stamped: 13-Sep-2023) [14]. Since the MSA sequences are not of the same length as the protein WT sequences we have to compute indices which WT positions to score. The WT position indices were derived via an *hmmer* hmmsearch of the respective HMM against the WT sequences, and the hit-ranges used as indices for the PSSM computation.

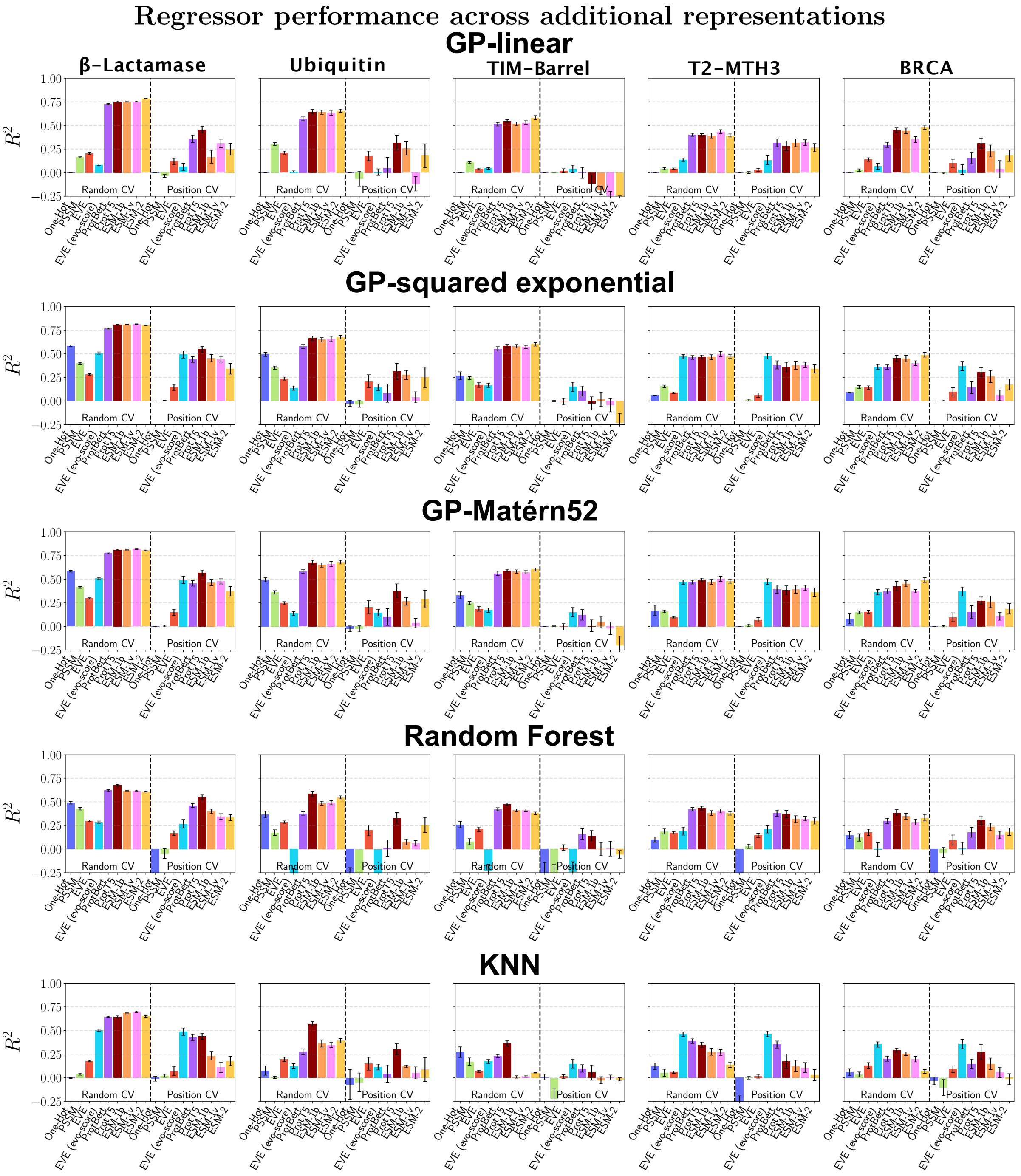

Fig D. Comparison of the performance (R^2) of individual regressors (rows) for splitting at random (10-fold) and by position (p=15) by embeddings ONE-HOT, PSSM, EVE, EVO-SCORE, PROTBERT, PROTT5, ESM-1B, ESM-1V, and ESM-2.

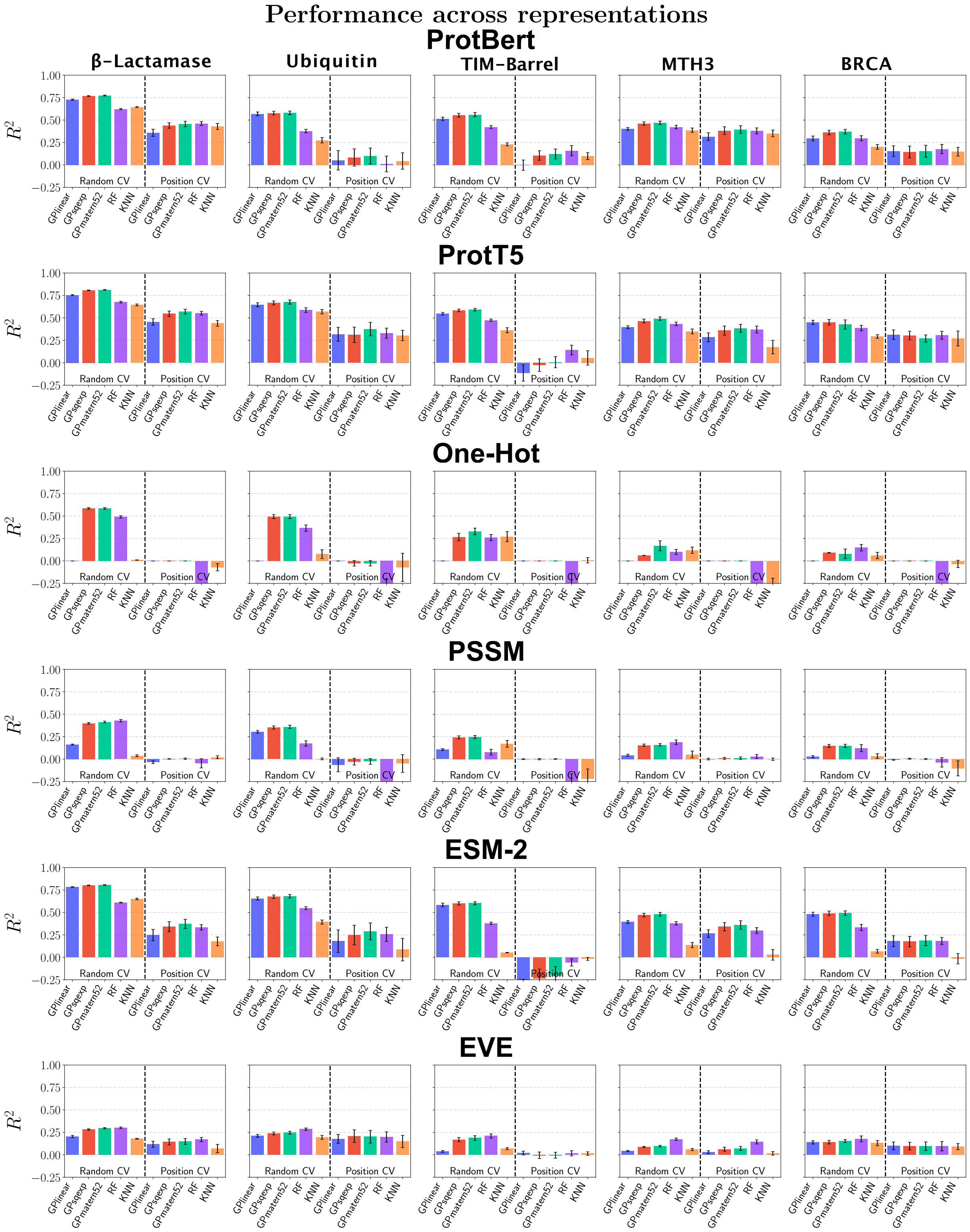

Fig E. Individual representation (rows) performance (R^2) across the available regressors (x-axis).

Fractional splitting results

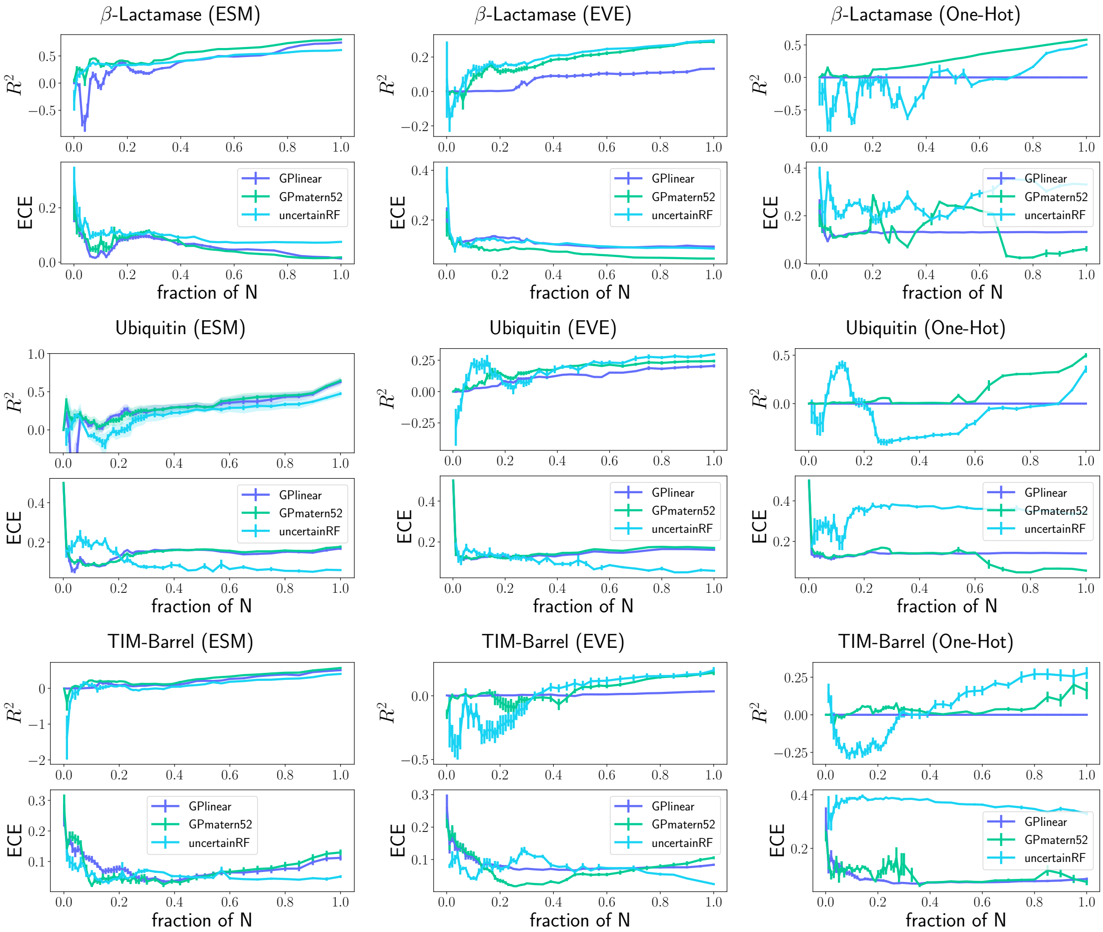

Fig F. Individual performance (R^2) and deviation from calibration (ECE) across fractions of available training data (splitting protocol, 5-fold CV per fraction), for β-Lactamase, Ubiquitin, TIM-Barrel, three different regressors (GP linear, GP Matérn$\frac{5}{2}$, and Random Forest with uncertainty estimate) on three different representations (ESM-1b, EVE, one-hot). The bars indicate std.err. across CV splits.

Note that for the β-Lactamase *uncertain RF* regressor experiments the optimal parameters from the random CV protocols were used for the second half of the data-fraction experiments across all representation, specifically splits $\{0.36, 0.39, \ldots\ 0.95, 1.0\}$ for computational feasibility. This only applies for the RF experiments.

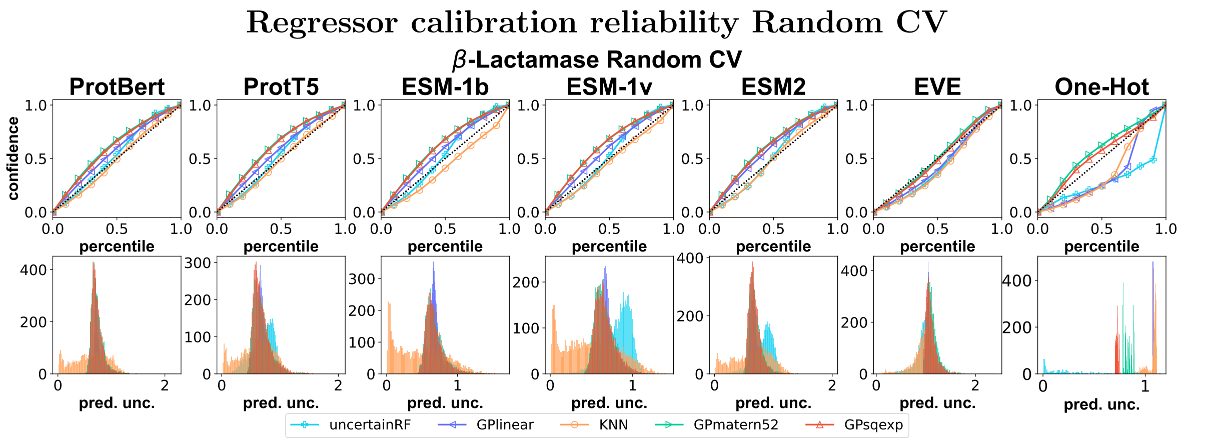

(a) Calibration by reliability curves (top) of β-Lactamase split at random and histogram of predictive uncertainties (bottom).

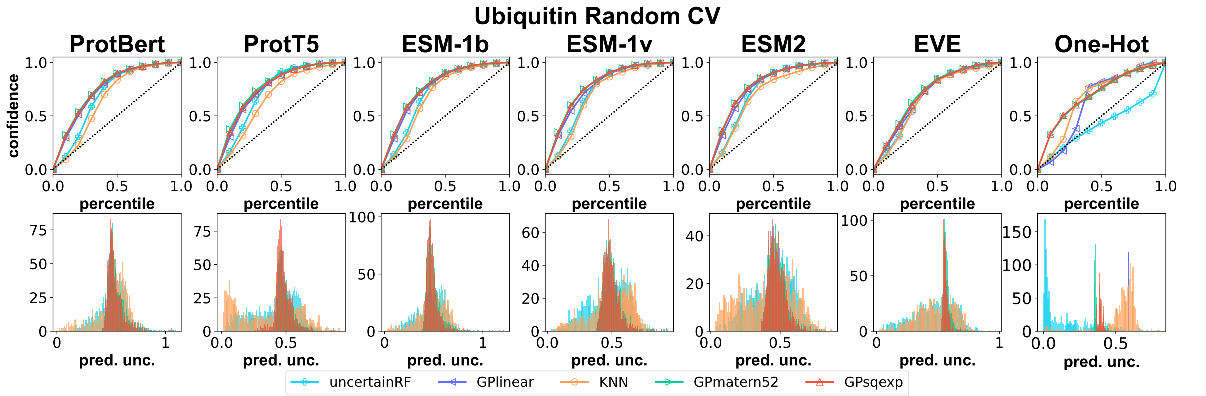

(b) Calibration by reliability curves (top) of Ubiquitin for splitting at random and histogram of predictive uncertainties (bottom).

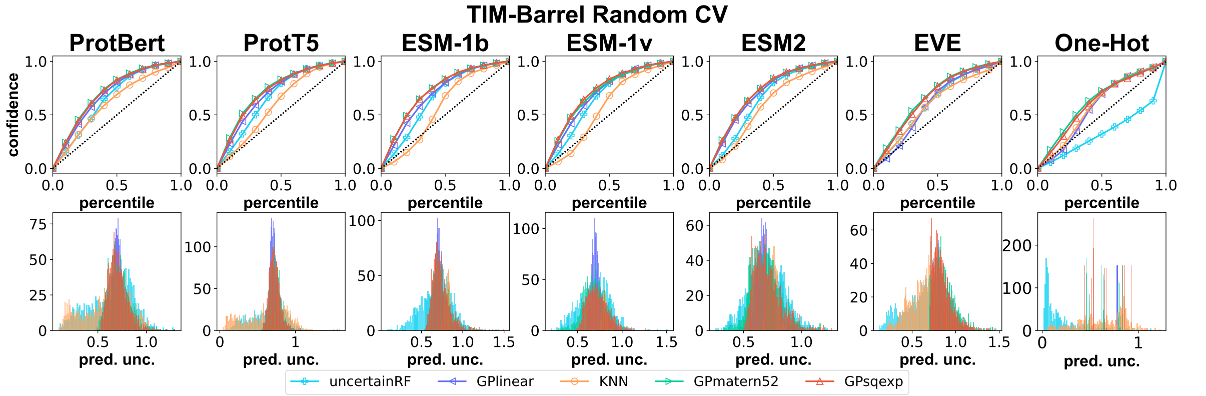

(c) Calibration by reliability curves (top) of TIM-Barrel for splitting at random and histogram of predictive uncertainties (bottom).

Fig G. Calibration of regressors by reliability curves for splitting by Random CV.

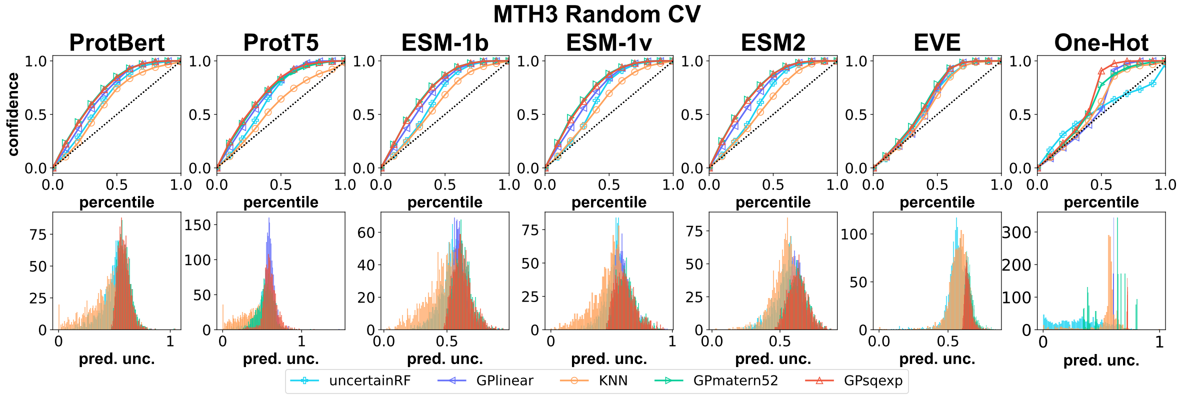

(a) Calibration by reliability curves (top) of T2-MTH for splitting at random and histogram of predictive uncertainties (bottom).

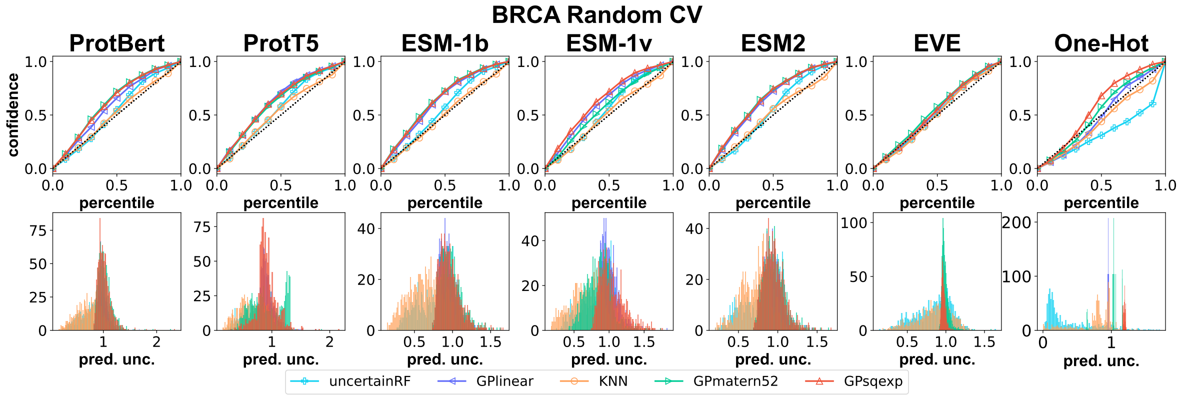

(b) Calibration by reliability curves (top) of BRCA1 for splitting at random and histogram of predictive uncertainties (bottom).

Fig H. Calibration of regressors by reliability curves for splitting by Random CV.

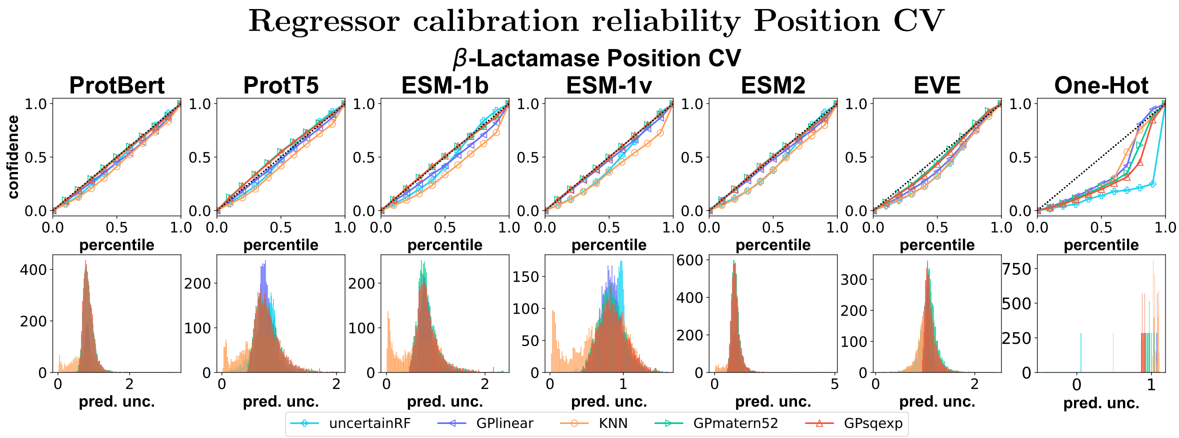

(a) Calibration by reliability curves (top) of β-Lactamase for splitting by position and histogram of predictive uncertainties (bottom).

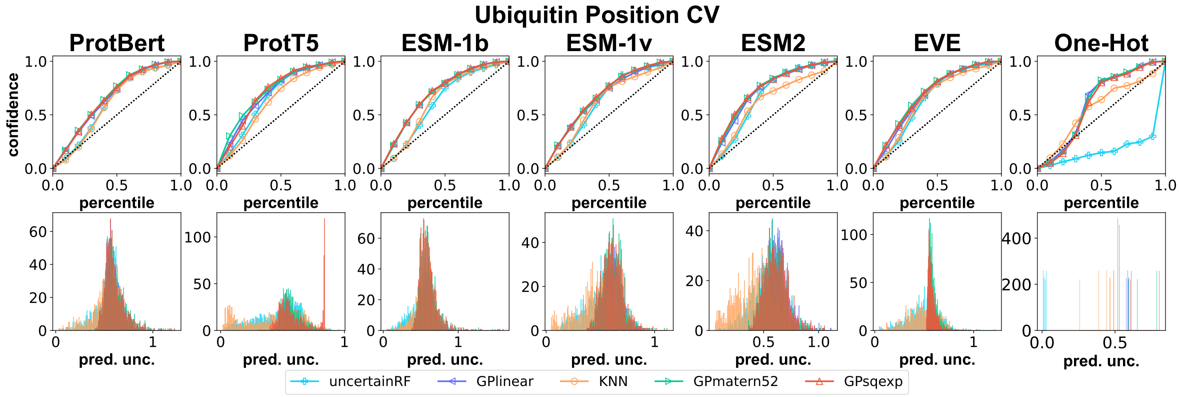

(b) Calibration by reliability curves (top) of Ubiquitin for splitting by position and histogram of predictive uncertainties (bottom).

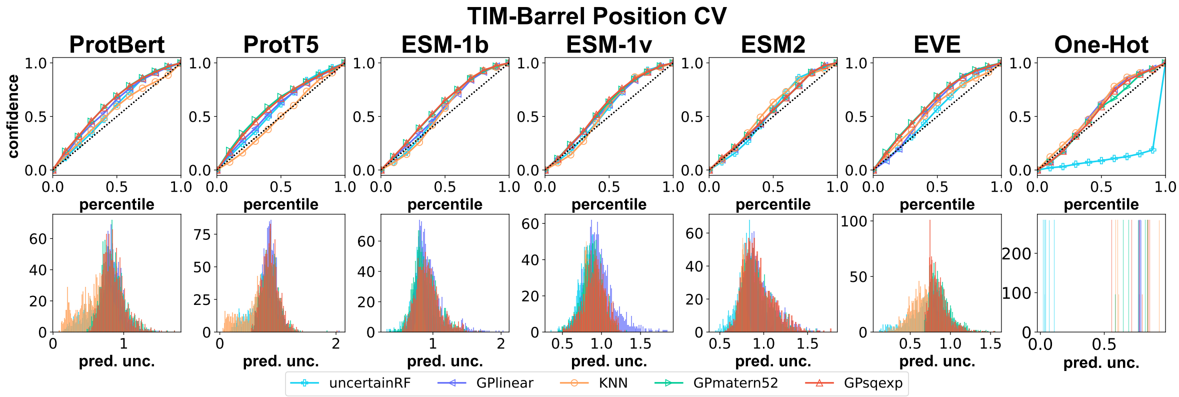

(c) Calibration by reliability curves (top) of TIM-Barrel for splitting by position and histogram of predictive uncertainties (bottom).

Fig I. Calibration of regressors by reliability curves for splitting by position.

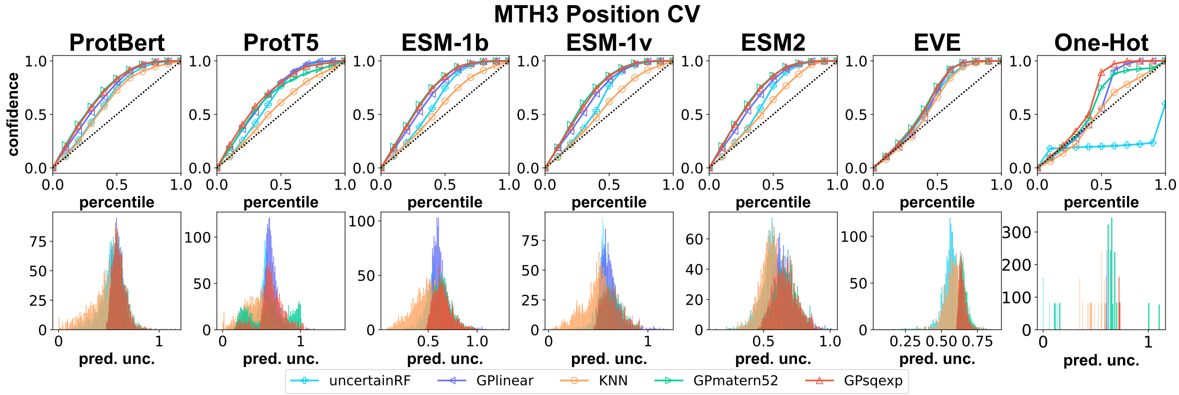

(a) Calibration by reliability curves (top) of T2-MTH for splitting by position and histogram of predictive uncertainties (bottom).

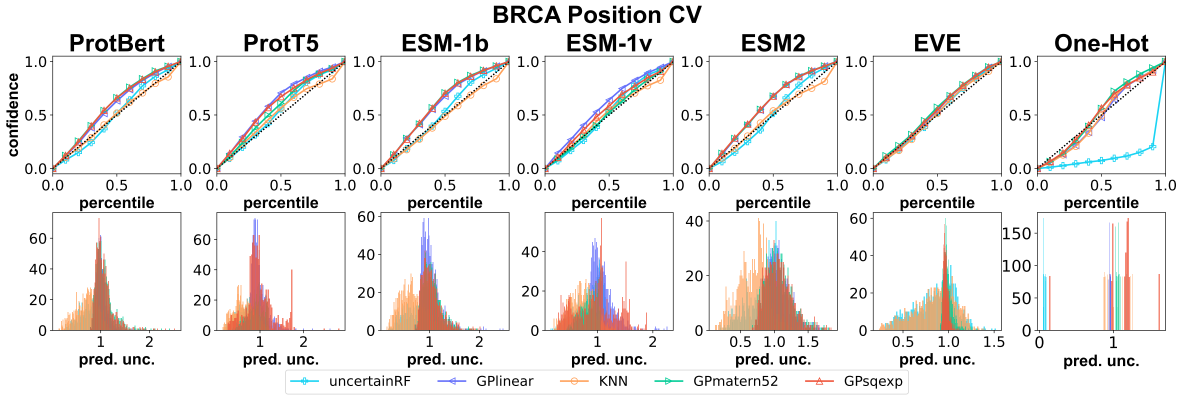

(b) Calibration by reliability curves (top) of BRCA1 for splitting by position and histogram of predictive uncertainties (bottom).

Fig J. Calibration of regressors by reliability curves for splitting by position.

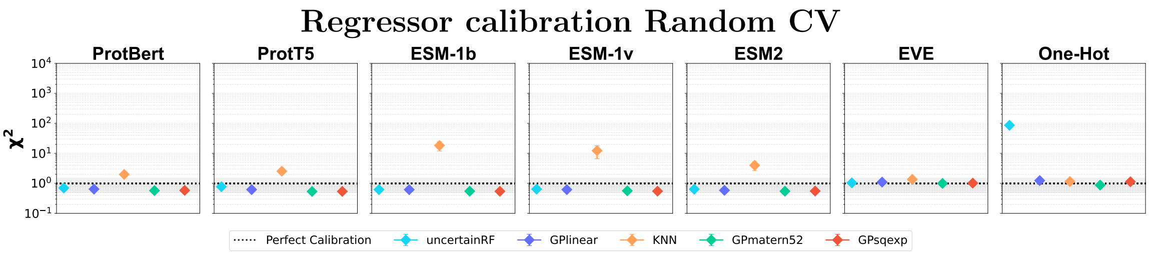

(a) χ^2 calibration of regressors on β-LACTAMASE when splitting at random.

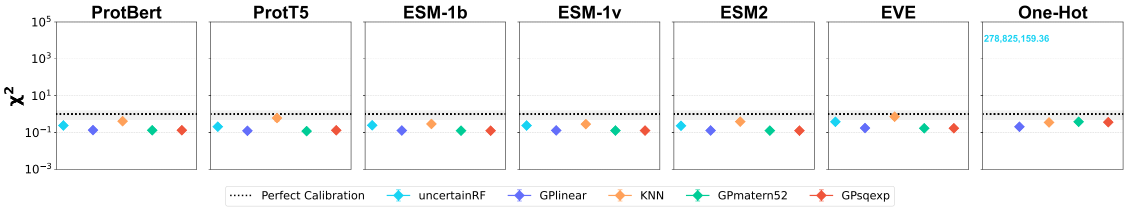

(b) χ^2 calibration of regressors on UBIQUITIN when splitting at random.

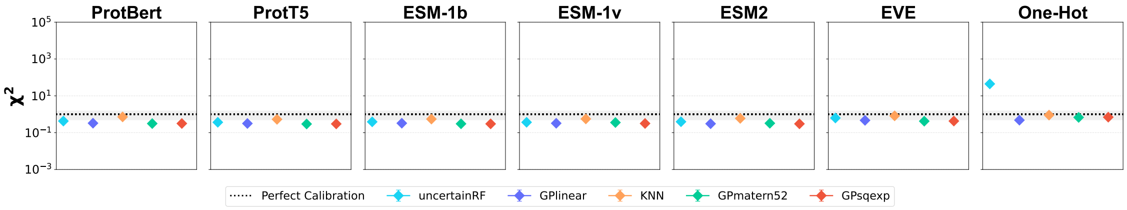

(c) χ^2 calibration of regressors on TIM-BARREL when splitting at random.

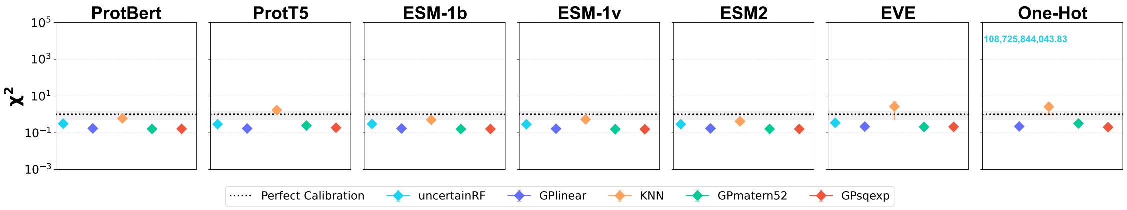

(d) χ^2 calibration of regressors on T2-MTH when splitting at random.

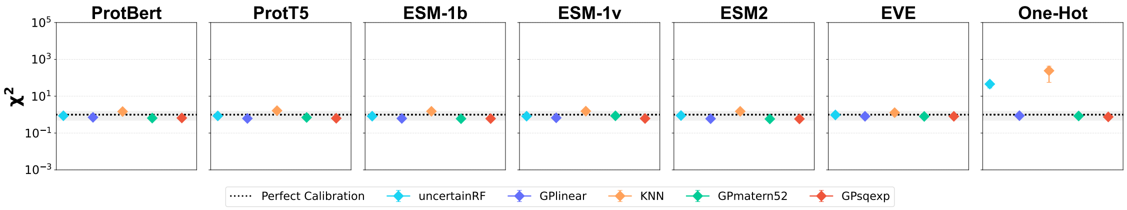

(e) χ^2 calibration of regressors on BRCA1 when splitting at random.

Fig K. Calibration of regressors by (reduced) χ^2 for splitting at random.

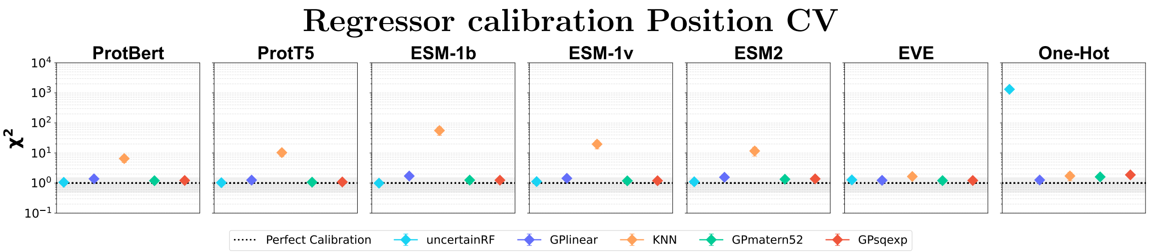

(a) χ^2 calibration of regressors on β-LACTAMASE when splitting by position.

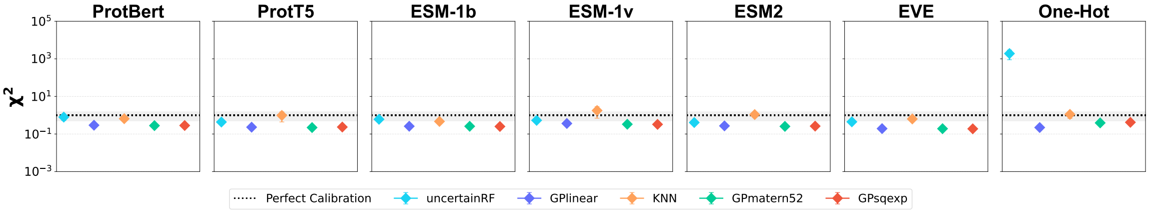

(b) χ^2 calibration of regressors on UBIQUITIN when splitting by position.

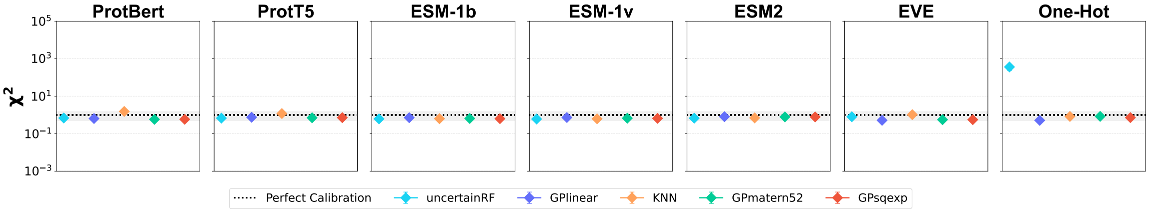

(c) χ^2 calibration of regressors on TIM-BARREL when splitting by position.

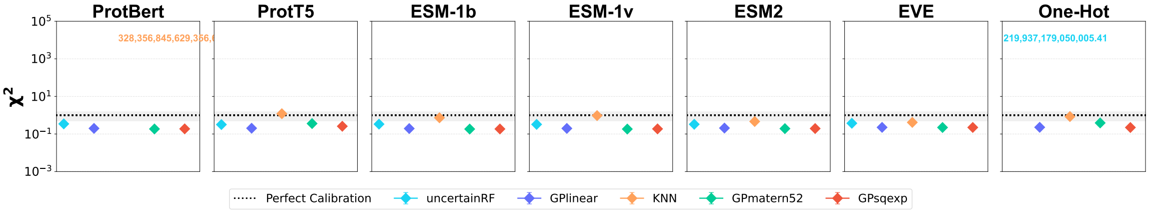

(d) χ^2 calibration of regressors on T2-MTH when splitting by position.

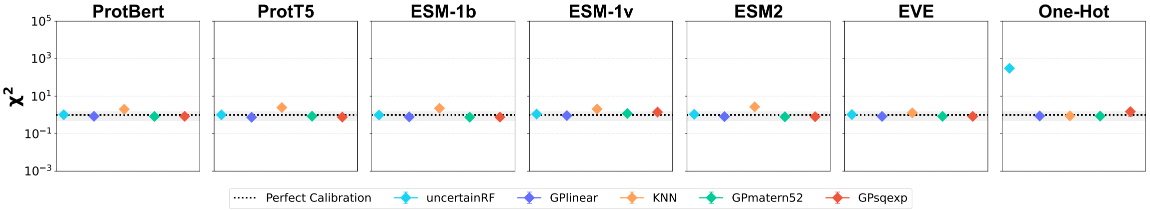

(e) χ^2 calibration of regressors on BRCA1 when splitting by position.

Fig L. Calibration of regressors by (reduced) χ^2 for splitting by position. In the case that the values are outside of the range (y-axis), the numerical value is displayed instead.

4 CONSIDERATIONS ON THE INDEPENDENCE OF PROTEIN VARIANTS

In addition to the described domains for protein variants, their distributions and properties the following situation arises.: Depending on the correlation of the positions and type of mutation multiple variants may or may *not* be considered *independent*. Let m_{A} and m_{B} be two mutations on a protein variant sequence S. Then $m_{\mathrm{A}} \perp\!\!\!\perp m_{\mathrm{B}}$ on S, iff

1. m_{A} is sufficiently distant to m_{B}, such that the residue properties do not impose on one another given the secondary and tertiary structure of the sequence, e.g. a distance around each residue of $r > 5$Å,
2. the evolutionary co-occurrence of the residues is not significant.

The latter point can be computed by the sequence's evolutionary statistical energy $E \in [0,1]$, and a dependency threshold $t \in [0,1]$ then

$$\Delta E(S \setminus m_{\mathrm{A}}, S \setminus m_{\mathrm{B}}) < t \tag{1}$$

(see (3) in [15]).
In contrast, two mutations on one sequence which are in close proximity and or have a high covariate dependency may be treated as one unit for downstream evaluation purposes.

5 MULTIMODALITY OF OBSERVATIONS

Sequences can be categorized into and *high* fitness ($Y^{(kM)^+} := \{\forall y \in Y^{(kM)} : y > t\}$) and *low* fitness ($Y^{(kM)^-} := \{Y^{(kM)} \backslash Y^{(kM)^+}\}$) relative to the initial wild-type observation or a threshold ($t \in \mathbb{R}$) of interest. Generally, for such data, we observe significantly more *low* variates: $|Y^{(kM)^+}| \ll |Y^{(kM)^-}|$ when higher order variants are considered. One can look at the modality in the observation distribution by a WT threshold (this applies particularly to Fig 6). To investigate this effect two scenarios are possible.: Either, we filter the available data to adapt the domain and task respective a functional threshold - selecting $\{(X_i, y_i) | y_i \geq \mathrm{t}\}$, where t is an expected threshold, e.g. the WT performance. Alternatively, we use all available data during training, but only measure performance on a subset; thus filter given the threshold mentioned above.

6 χ^2 CALIBRATION

When considering the (reduced) χ^2 statistic for the goodness of fit, the normalization constant can additionally be extended to account for the degrees of freedom of the regressor. This is well defined for the Random Forest [16], however less so for non-parametric models such as the Gaussian Process regressors. Therefore the metric in its presented equation encapsulates also the inherent model complexity.
To compute the metric in practice we make the assumption of independence between predictions under the model, specifically $\{\hat{\sigma}_i^2\}_{i=1..N}$ such that we can build a covariance matrix from the diagonal of the predictive variances and solve by cholesky factorization - as done in [17]. This is equivalent to the previously stated sum of residuals (normalized by the predictive variances) under the previously stated assumptions.

7 FRACTIONAL SPLITTING

The performance of the Random Forest and GP (Matérn$\frac{5}{2}$) shows a monotonic increase over the fractions (for the majority of data-sets and representations); while all regressors show performance fluctuations with low sequence numbers. All methods steadily improve after approx.

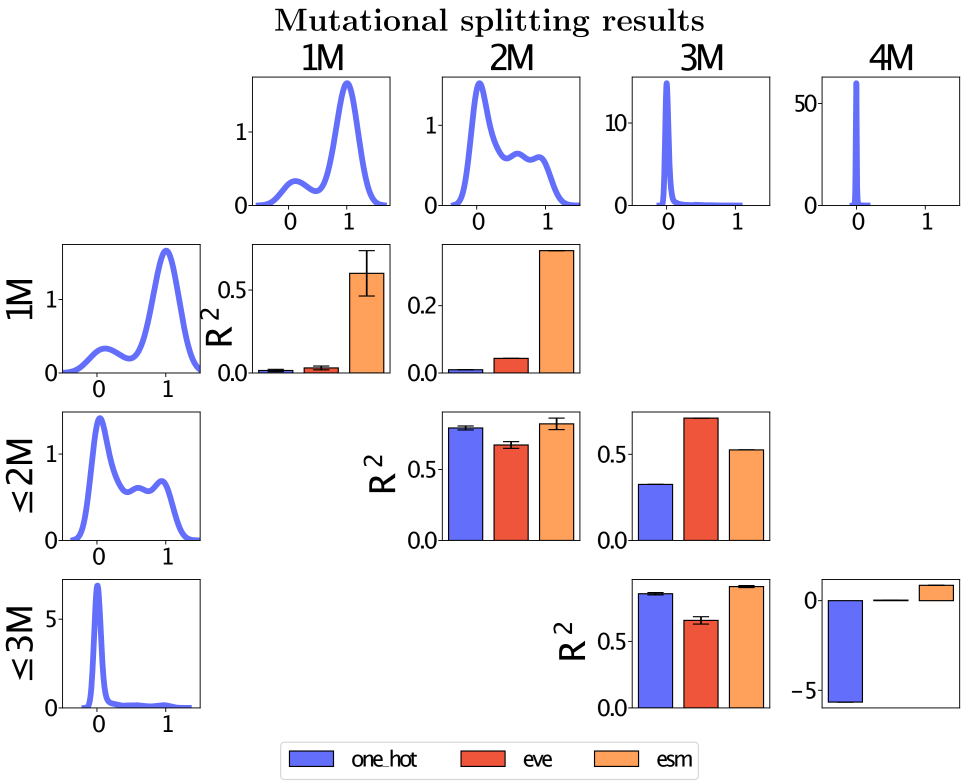

Fig M. R^2 metric on *mutation-degree* protocol on GP (Matérn$\frac{5}{2}$) represented by ESM-1B. Functional observations of PARD-ANTITOXIN are density curves in first row and column. We assess the adjusted R^2 score. The training domains are listed on rows and testing domains are columns, such that diagonals display in-domain performance, and off diagonal extrapolation performance. To assess in-domain performance a standard 5-fold CV protocol was used.

30% available training data. All models appear sufficiently well calibrated. Only on the ONE-HOT representation the ECE values of the GPs are significantly lower compared to the RF. The performance suggests GP-Matérn and RF to perform well, both on individual fractions. We note that the performance of all methods on the ESM-1B representation is comparable to that of the shown PROTBERT model. In contrast to the language models, the regressors'performance deteriorate on the ONE-HOT embedding of β-LACTAMASE.

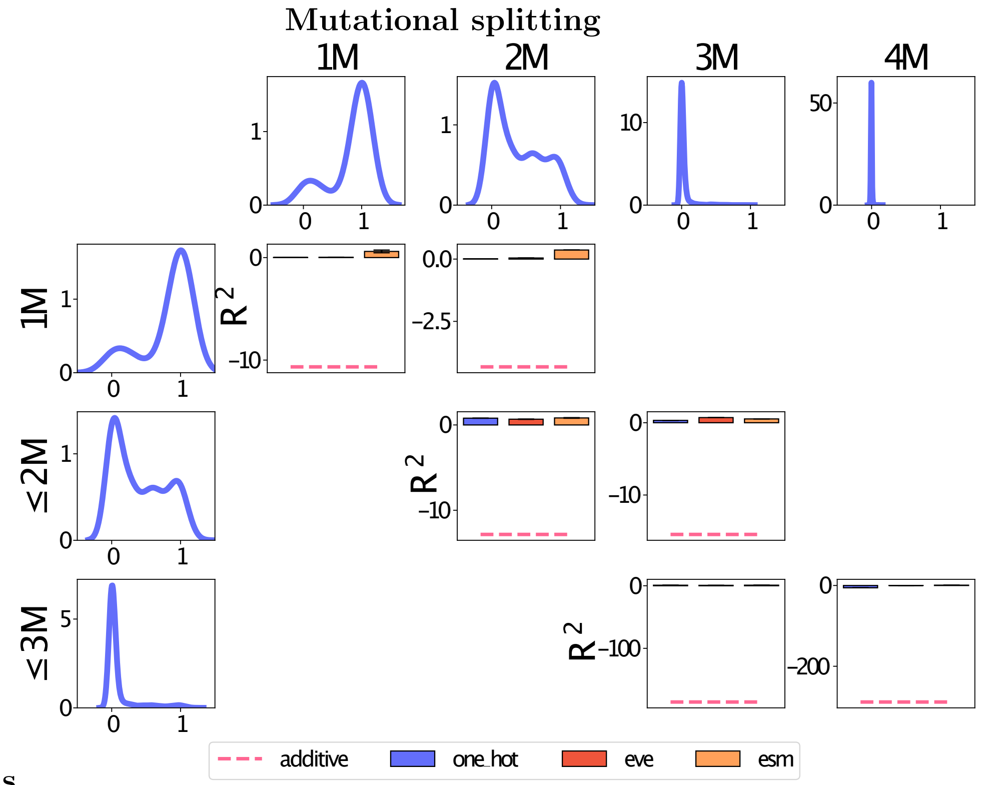

Results

Fig N. R^2 metric on *mutation-degree* protocol on GP (Matérn$\frac{5}{2}$) represented by ESM-1B including *additive mutations* as reference baseline. Functional observations of ParD-antitoxin are density curves in first row and column. We assess the adjusted R^2 score. The training domains are listed on rows and testing domains are columns, such that diagonals display in-domain performance, and off diagonal extrapolation performance. To assess in-domain performance a standard 5-fold CV protocol was used.

8 CV PROTOCOL PARAMETERS

A generalization protocol test-error assessment is subject to the amount of splits conducted and how much data is available per split. This applies specifically to the Random CV protocol and the Positional splitting protocol. For the Random CV this is the k parameter, i.e. 3-,5-,10-fold cross validation sampled uniformly at random, and for the Position CV this is the p parameter, i.e. eliminating ranges of size 5 or 25.

9 OPTIMIZATION RESULTS

After an initial random selection, we add the next best candidate sequence by its highest expected reward from the predictions of the regressor, and then retrain the model, with the new observation in the data-set. We have two reference baselines: 1) selecting sequences at random, and 2) a static protocol, which iterates over a fixed score-ranking proposal of an unsupervised model.[1] At each step we report the function value of the currently selected best sequence candidate, the average across all observations, and the cumulative regret (Fig S)). The results on the ProtBert representation show that the GP based models find the optimal candidate after the fewest observations (see Fig S). The static, unsupervised baseline demonstrates strong performance in turn of the obtained mean and regret scores, but ultimately uses more iterations to find the optimal solution. The reason for this behavior is that the EVE model orders by evo-score, such that low value observations are placed within the first 500 iterations, while the regressors do not perform such an explicit ordering. Instead, the optimization method iteratively scores the variants by its *acquisition function* and updates the scores at each iteration. Though the Random Forest method has previously shown comparatively high accuracy, the absolute best value is not found within the allotted budget. The predictive uncertainties for the random forest stay constant during extrapolation respective the expected ensemble variance of the closest known value. This stands in contrast to the GP uncertainties, which increase for extrapolation predictions. This leads to less exploration of sequences by the RF, which prohibits us to find the best candidate. On the other hand, the properties of the GPs allow for exploration in regions of higher uncertainties. We note that the performance is similar between ProtBert and the ESM-1b representation, with the best observations being found after the evo-scores (over 300 steps) instead (see SI Fig S). These additional results show that we cannot quantify the exact expected optimization performance, given a cumulative metric and calibration, but can only obtain an approximate order for the assessed methods and are dependent on the representation.

[1] In our case this is the evolutionary scoring of the EVE model, which is the relative loss-bound across 2000 samples.

Table B. Train- Test-Set sizes Random CV and Positional CV.
Overview of the sizes of the data sets for [*training* ; *test*] for the initial benchmarking protocol splits (rows) - standard 10-fold Cross Validation and splitting by positions. Note that due to the difference in length between the protein sequences there is a different amount of splits available for each. The representation column accounts for the ONE-HOT, PROTBERT, ESM-1B, EVE embeddings, while eve-density is a subset of EVE and for some proteins there are less sequences available, compared to the total dataset.

		β-LACTAMASE		UBIQUITIN		TIM-BARREL		T2-MTH		BRCA1	
		representations	eve_density	representations	eve_density	representations	eve_density	representations	eve_density	representations	eve_density
**Random CV**	**0**	[4309;479]	[4309;479]	[1074;120]	[1043;116]	[1367;152]	[1367;152]	[1547;172]	[1547;172]	[934;104]	[934;104]
	**1**	[4309;479]	[4309;479]	[1074;120]	[1043;116]	[1367;152]	[1367;152]	[1547;172]	[1547;172]	[934;104]	[934;104]
	**2**	[4309;479]	[4309;479]	[1074;120]	[1043;116]	[1367;152]	[1367;152]	[1547;172]	[1547;172]	[934;104]	[934;104]
	**3**	[4309;479]	[4309;479]	[1074;120]	[1043;116]	[1367;152]	[1367;152]	[1547;172]	[1547;172]	[934;104]	[934;104]
	**4**	[4309;479]	[4309;479]	[1075;119]	[1043;116]	[1367;152]	[1367;152]	[1547;172]	[1547;172]	[934;104]	[934;104]
	**5**	[4309;479]	[4309;479]	[1075;119]	[1043;116]	[1367;152]	[1367;152]	[1547;172]	[1547;172]	[934;104]	[934;104]
	**6**	[4309;479]	[4309;479]	[1075;119]	[1043;116]	[1367;152]	[1367;152]	[1547;172]	[1547;172]	[934;104]	[934;104]
	**7**	[4309;479]	[4309;479]	[1075;119]	[1043;116]	[1367;152]	[1367;152]	[1547;172]	[1547;172]	[934;104]	[934;104]
	**8**	[4310;478]	[4310;478]	[1075;119]	[1043;116]	[1367;152]	[1367;152]	[1547;172]	[1547;172]	[935;103]	[935;103]
	**9**	[4310;478]	[4310;478]	[1075;119]	[1044;115]	[1368;151]	[1368;151]	[1548;171]	[1548;171]	[935;103]	[935;103]
**Position CV p=15**	**0**	[4427;285]	[4427;285]	[898;220]	[863;220]	[1159;284]	[1159;284]	[1620;79]	[1620;79]	[933;82]	[933;82]
	**1**	[4351;285]	[4351;285]	[797;258]	[762;258]	[1082;285]	[1082;285]	[1597;79]	[1597;79]	[912;82]	[912;82]
	**2**	[4351;285]	[4351;285]	[829;260]	[794;260]	[1082;285]	[1082;285]	[1596;81]	[1596;81]	[904;87]	[904;87]
	**3**	[4351;285]	[4351;285]	[824;230]	[789;230]	[1082;285]	[1082;285]	[1591;83]	[1591;83]	[914;76]	[914;76]
	**4**	[4351;285]	[4351;285]	[900;226]	[900;191]	[1082;285]	[1082;285]	[1588;85]	[1588;85]	[982;36]	[982;36]
	**5**	[4351;285]	[4351;285]			[1348;95]	[1348;95]	[1596;82]	[1596;82]	[911;90]	[911;90]
	**6**	[4351;285]	[4351;285]					[1595;81]	[1595;81]	[912;78]	[912;78]
	**7**	[4351;285]	[4351;285]					[1588;84]	[1588;84]	[912;83]	[912;83]
	**8**	[4484;228]	[4484;228]					[1595;82]	[1595;82]	[905;86]	[905;86]
	**9**	[4351;285]	[4351;285]					[1599;77]	[1599;77]	[905;87]	[905;87]
	**10**	[4351;285]	[4351;285]					[1599;77]	[1599;77]	[905;84]	[905;84]
	**11**	[4351;285]	[4351;285]					[1589;83]	[1589;83]	[910;84]	[910;84]
	**12**	[4351;285]	[4351;285]					[1597;79]	[1597;79]	[912;83]	[912;83]
	**13**	[4351;285]	[4351;285]					[1600;80]	[1600;80]		
	**14**	[4351;285]	[4351;285]					[1679;18]	[1679;18]		
	**15**	[4351;285]	[4351;285]					[1592;83]	[1592;83]		
	**16**	[4351;285]	[4351;285]					[1602;78]	[1602;78]		
	**17**							[1599;77]	[1599;77]		
	**18**							[1593;82]	[1593;82]		
	**19**							[1592;82]	[1592;82]		
	**20**							[1591;82]	[1591;82]		
	**21**							[1592;85]	[1592;85]		

Table C. Train- Test-Set sizes mutational CV.
Overview of the sizes of the data sets for [*training* ; *test*] for each mutational split (rows). Intra-domain assessment ($1 \to 1$, $2 \to 2$, $3 \to 3$) is standard 5-fold cross-validation. Across domains (extrapolation) of mutations compared to the WT are $1 \to 2$, $\leq 2 \to 3$, $\leq 3 \to 4$.

		PARD-ANTITOXIN
**BioSplitter1_1**	**0**	[31;7]
	**1**	[31;7]
	**2**	[31;7]
	**3**	[31;7]
	**4**	[31;7]
**BioSplitter1_2**	**0**	[38;499]
**BioSplitter2_2**	**0**	[438;99]
	**1**	[438;99]
	**2**	[438;99]
	**3**	[438;99]
	**4**	[438;99]
**BioSplitter2_3**	**0**	[537;2798]
**BioSplitter3_3**	**0**	[2776;559]
	**1**	[2776;559]
	**2**	[2776;559]
	**3**	[2776;559]
	**4**	[2776;559]
**BioSplitter3_4**	**0**	[3335;5858]

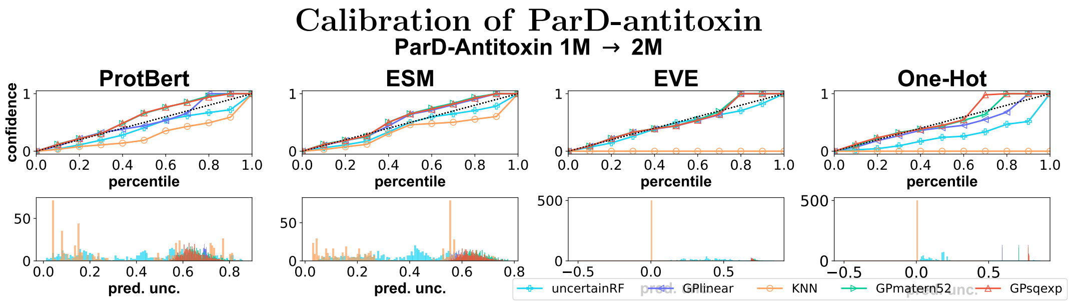

(a) Calibration of ParD-antitoxin (top) w.r.t. perfect calibration (dotted diagonal) and count of predictive variances (bottom) for the 1M to 2M extrapolation task.

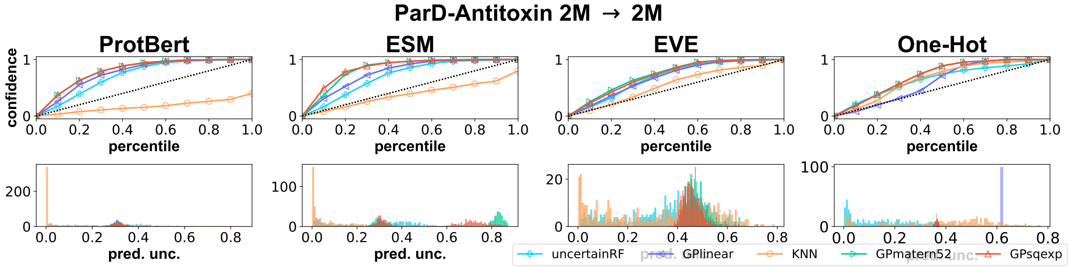

(b) Calibration of ParD-antitoxin (top) w.r.t. perfect calibration (dotted diagonal) and count of predictive variances (bottom) for the 2M to 2M intrapolation (5-fold CV) task.

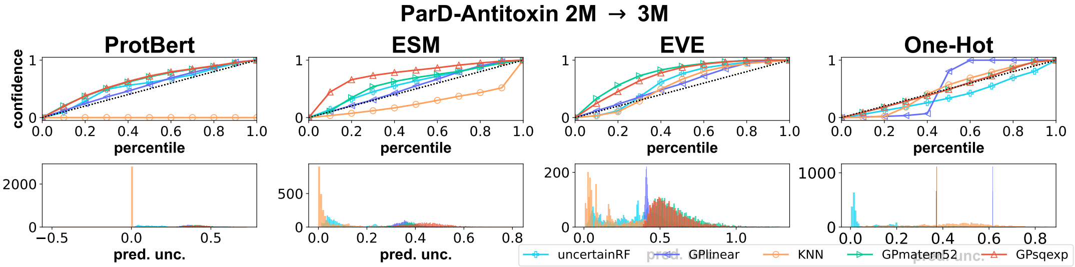

(c) Calibration of ParD-antitoxin (top) w.r.t. perfect calibration (dotted diagonal) and count of predictive variances (bottom) for the 2M to 3M extrapolation task.

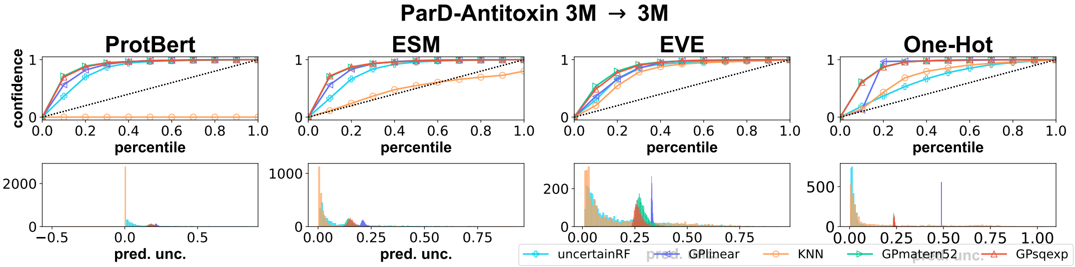

(d) Calibration of ParD-antitoxin (top) w.r.t. perfect calibration (dotted diagonal) and count of predictive variances (bottom) for the 3M to 3M intrapolation task.

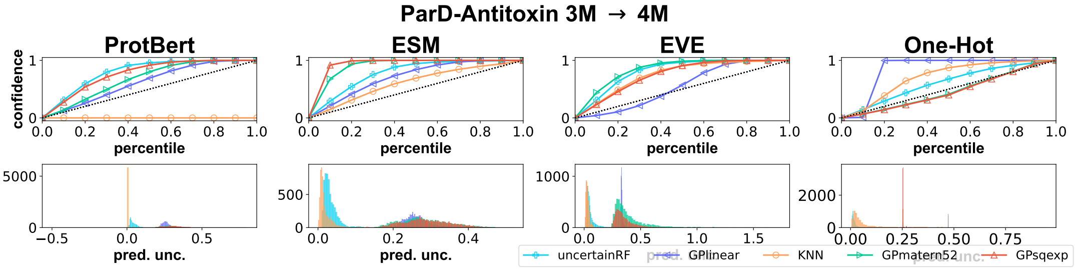

(e) Calibration of ParD-antitoxin (top) w.r.t. perfect calibration (dotted diagonal) and count of predictive variances (bottom) for the 3M to 4M extrapolation task.

Fig O. Calibration overview across extra- and intra-polation task assessments of ParD-antitoxin across representations (columns). Note the peaked predictive uncertainties (constant confidence curves) for KNN regressors with one neighbor predictions.

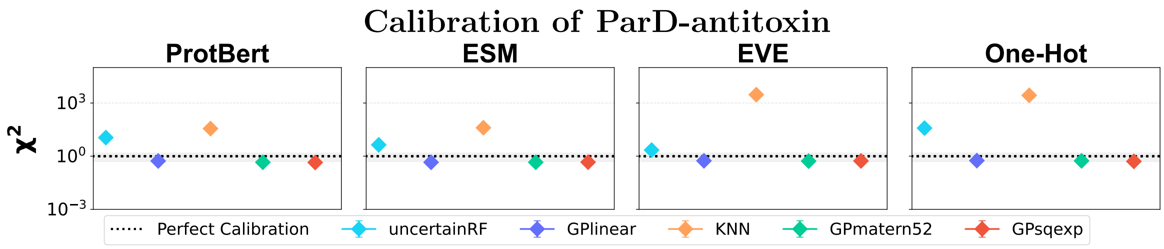

(a) Calibration by χ^2 assessment of ParD-antitoxin (top) w.r.t. perfect calibration (dotted line and grey region) for the 1M to 2M extrapolation task.

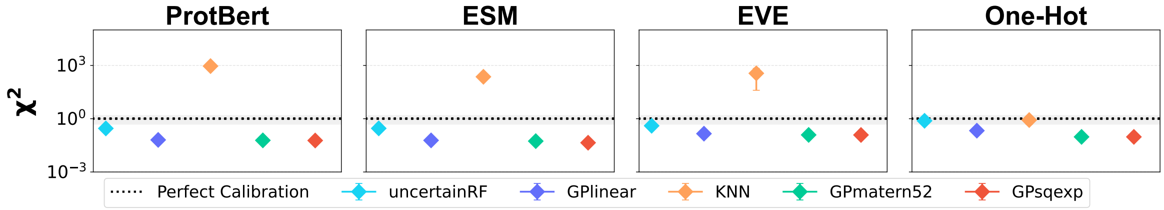

(b) Calibration by χ^2 assessment of ParD-antitoxin (top) w.r.t. perfect calibration (dotted line and grey region) for the 2M to 2M intrapolation task.

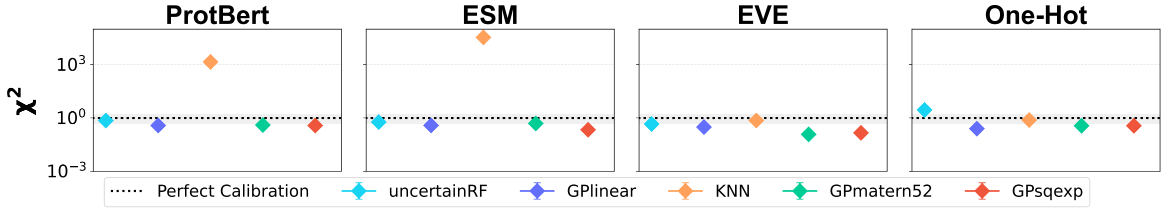

(c) Calibration by χ^2 assessment of ParD-antitoxin (top) w.r.t. perfect calibration (dotted line and grey region) for the 2M to 3M extrapolation task.

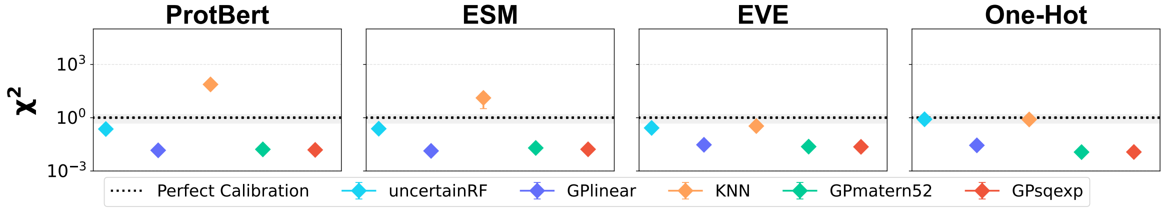

(d) Calibration by χ^2 assessment of ParD-antitoxin (top) w.r.t. perfect calibration (dotted line and grey region) for the 3M to 3M intrapolation task.

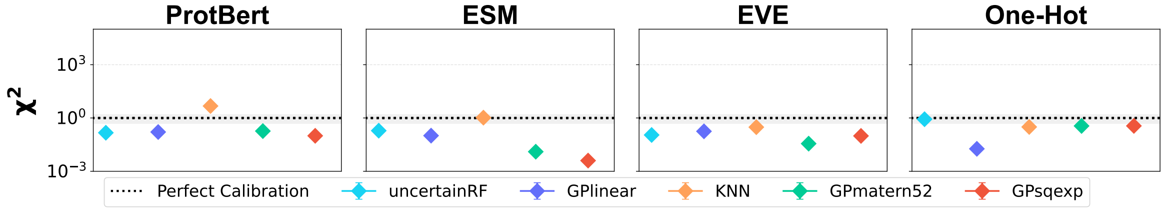

(e) Calibration by χ^2 assessment of ParD-antitoxin (top) w.r.t. perfect calibration (dotted line and grey region) for the 3M to 4M extrapolation task.

Fig P. χ^2 calibration overview across extra- and intrapolation task assessments of ParD-antitoxin across representations (columns).

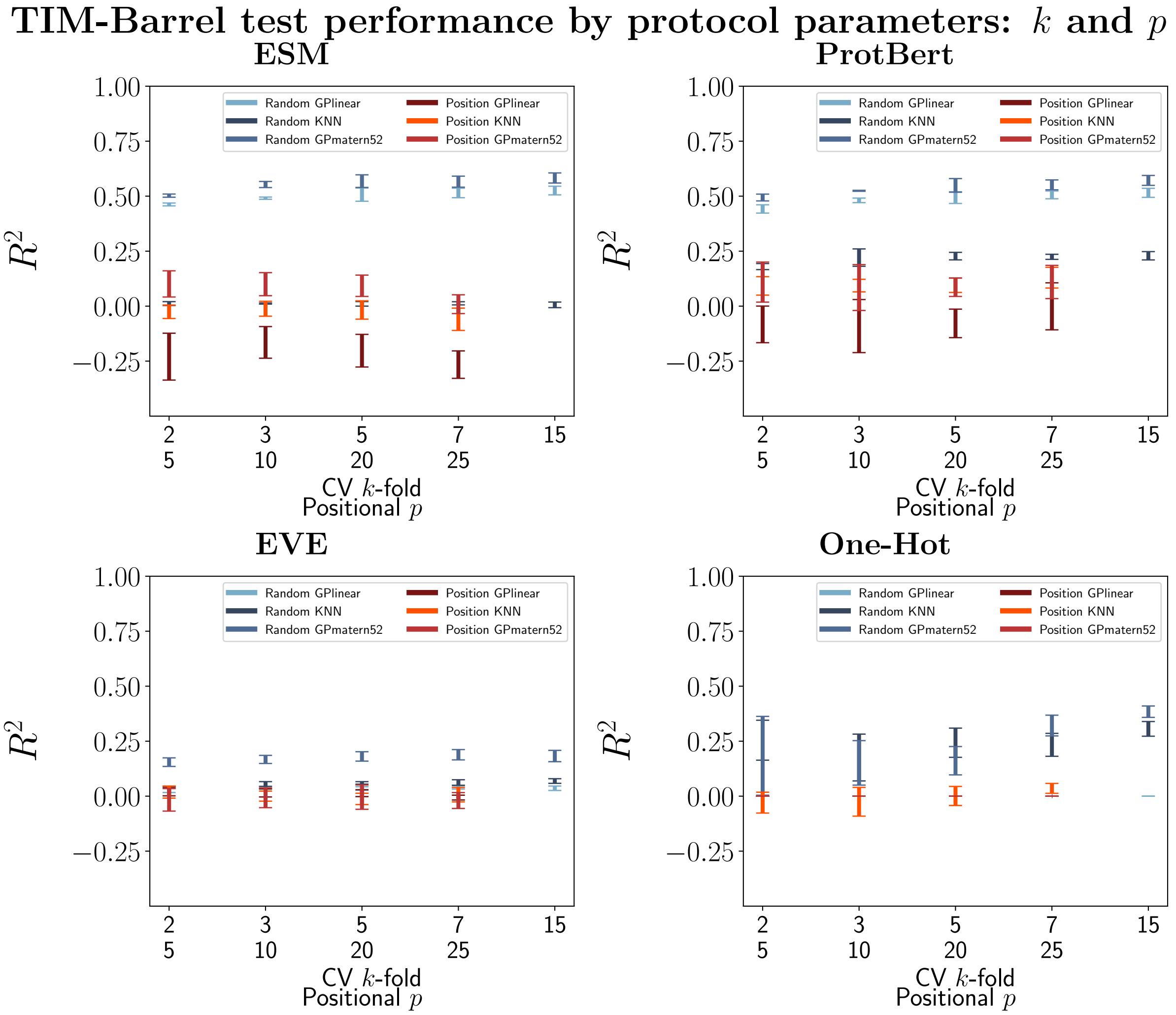

Fig Q. We fit a subset of regressors on the TIM-Barrel dataset and consider the test error (y-axis) by accuracy ($R2$) against the number of splits of the protocol. We do this for $k = 2, 3, 5, 7, 15$ (Random CV) and $p = 5, 10, 20, 25$ (Positional CV). We observe no significant changes in the expected performance.

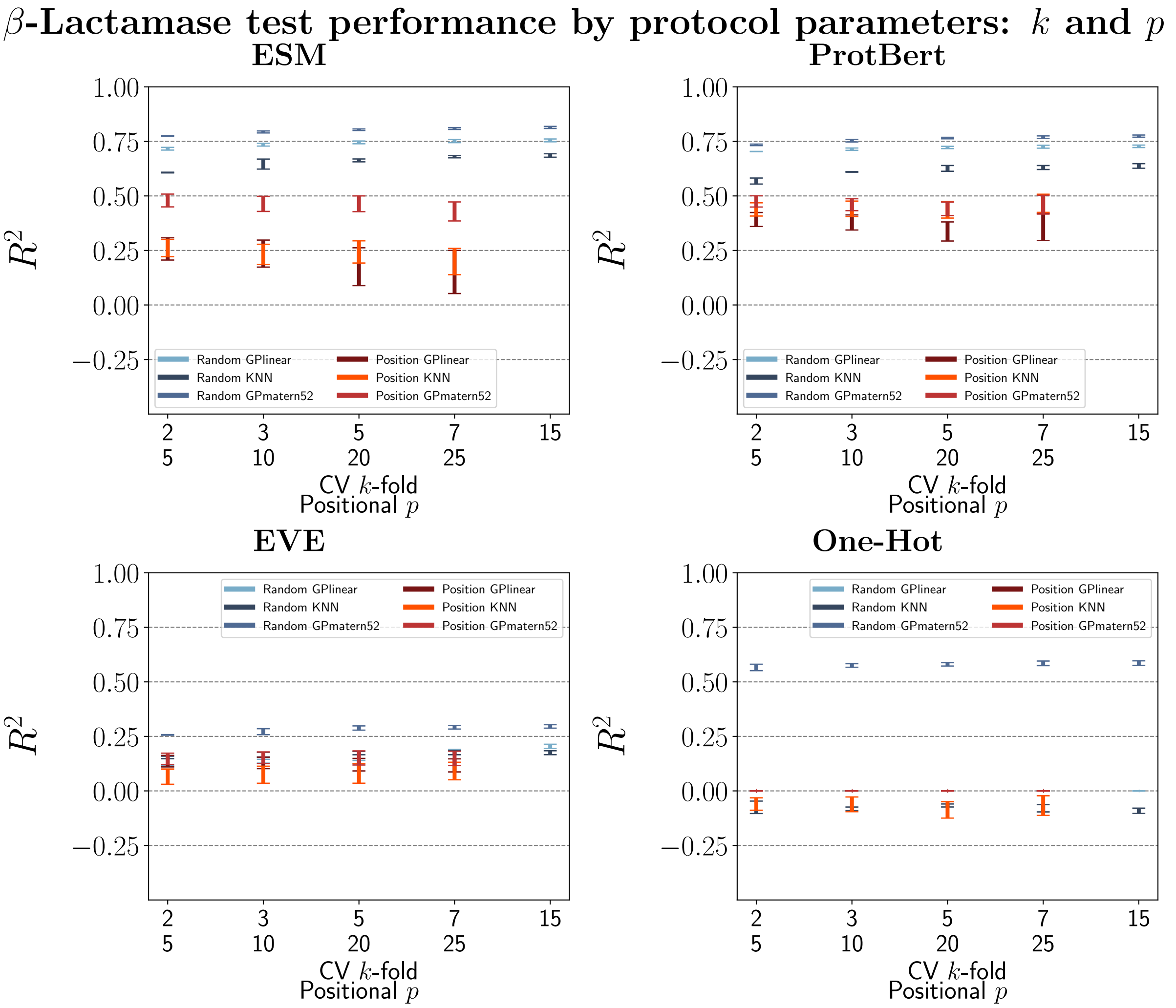

Fig R. We fit a subset of regressors on the β-LACTAMASE dataset and consider the test error (y-axis) by accuracy ($R2$) against the number of splits of the protocol. We do this for $k = \{2, 3, 5, 7, 15\}$ (Random CV) and $p = \{5, 10, 20, 25\}$ (Positional CV) to provide an addition to the previously discussed results are for $p = 15$ and $k = 10$. We observe no significant changes in the expected test performance.

Optimization of β-Lactamase

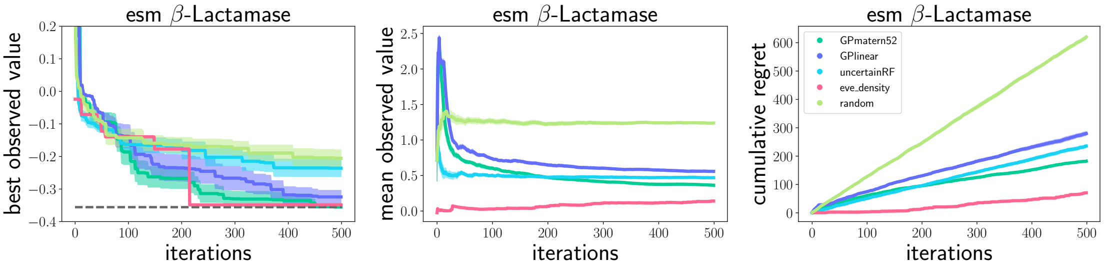

(a) Optimization experiment results of β-Lactamase on ESM-1B w.r.t. best observed value (left), mean value (center), and cumulative regret (right).

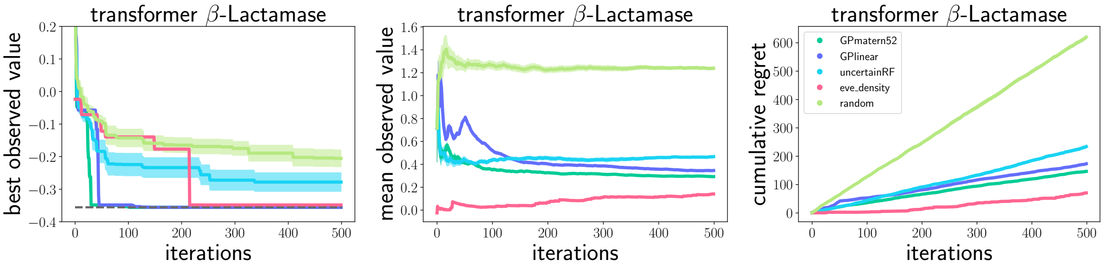

(b) Optimization experiment results of β-Lactamase on PROTBERT w.r.t. best observed value (left), mean value (center), and cumulative regret (right).

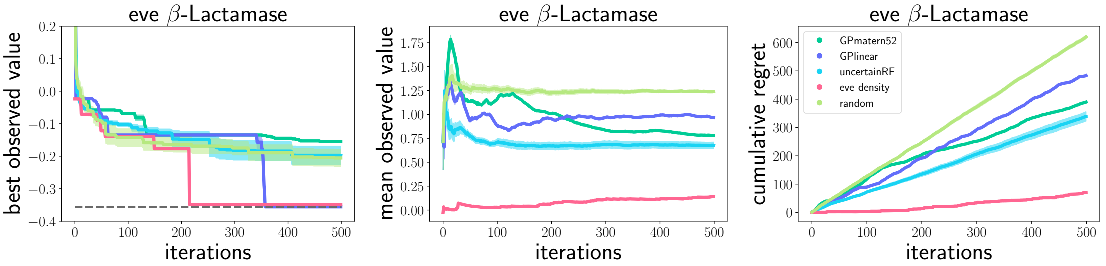

(c) Optimization experiment results of β-Lactamase on EVE w.r.t. best observed value (left), mean value (center), and cumulative regret (right).

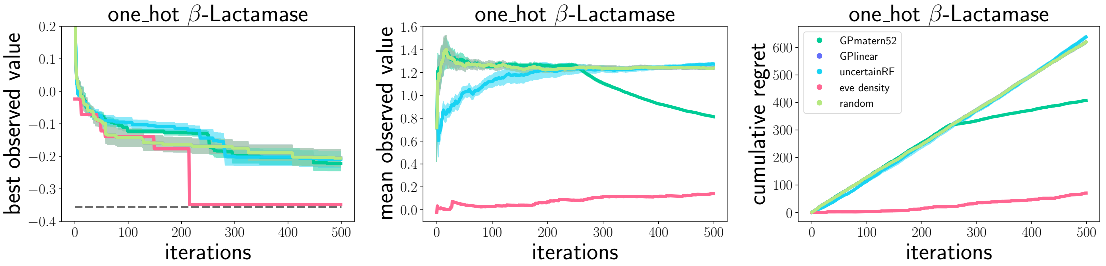

(d) Optimization experiment results of β-Lactamase on ONE-HOT w.r.t. best observed value (left), mean value (center), and cumulative regret (right).

Fig S. Optimization as sequence selection with a budget of 500 steps. Across selected sequence candidates the best values (left), mean prediction (middle) and cumulative regret (right). For each method the mean prediction (bold line) from 10 different random seed runs, and standard error across (shaded regions). The reference baselines are randomly selecting observations (light green), and iterating over the ranked sequences by EVE scoring (pink). The best possible value in the setup is the dashed grey line (left), which is found by the GP models and scored ranking within the allotted budget.

Optimization of Ubiquitin

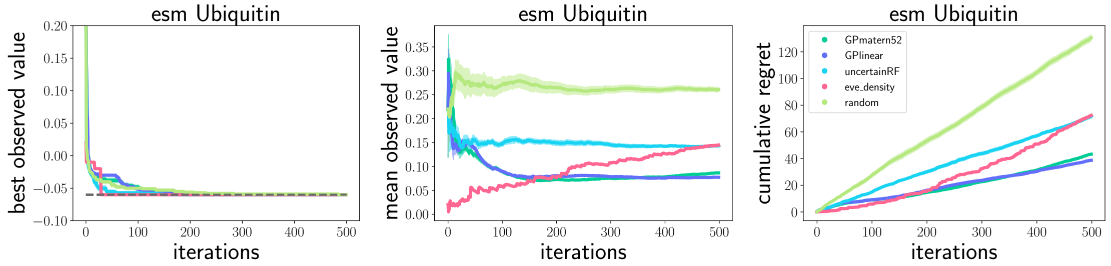

(a) Optimization experiment results of UBIQUITIN on ESM-1B w.r.t. best observed value (left), mean value (center), and cumulative regret (right).

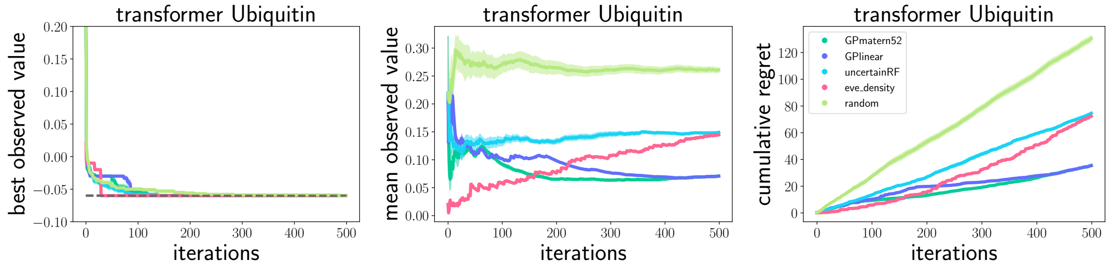

(b) Optimization experiment results of UBIQUITIN on PROTBERT w.r.t. best observed value (left), mean value (center), and cumulative regret (right).

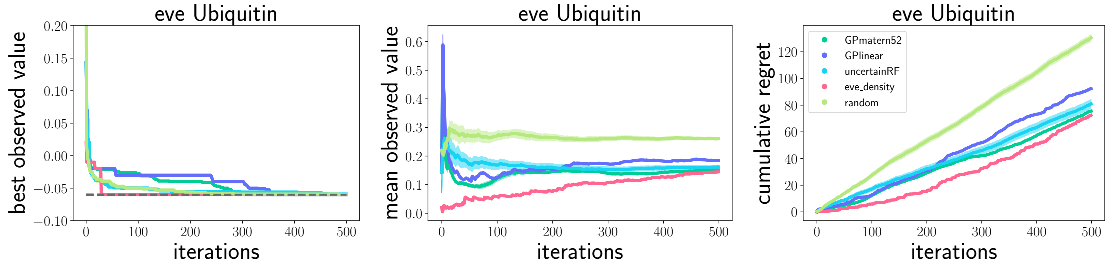

(c) Optimization experiment results of UBIQUITIN on EVE w.r.t. best observed value (left), mean value (center), and cumulative regret (right).

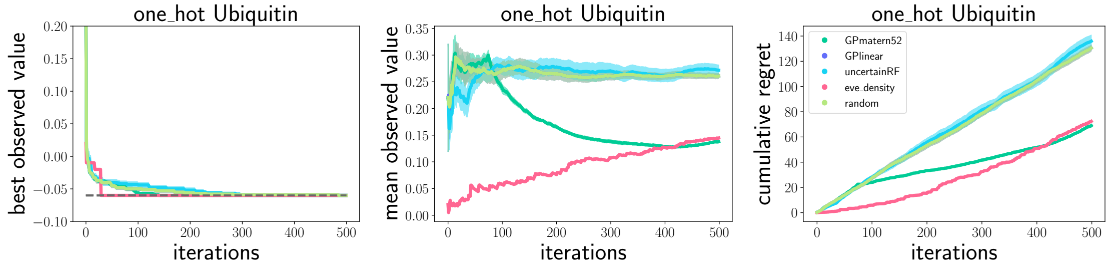

(d) Optimization experiment results of UBIQUITINon ONE-HOT w.r.t. best observed value (left), mean value (center), and cumulative regret (right).

Fig T. Optimization as sequence selection with a budget of 500 steps. Across selected sequence candidates the best values (left), mean prediction (middle) and cumulative regret (right). For each method the mean prediction (bold line) from 10 different random seed runs, and standard error across (shaded regions). The reference baselines are randomly selecting observations (light green), and iterating over the ranked sequences by EVE scoring (pink). The best possible value in the setup is the dashed grey line (left), which is found by all regressors and scored ranking within the allotted budget.

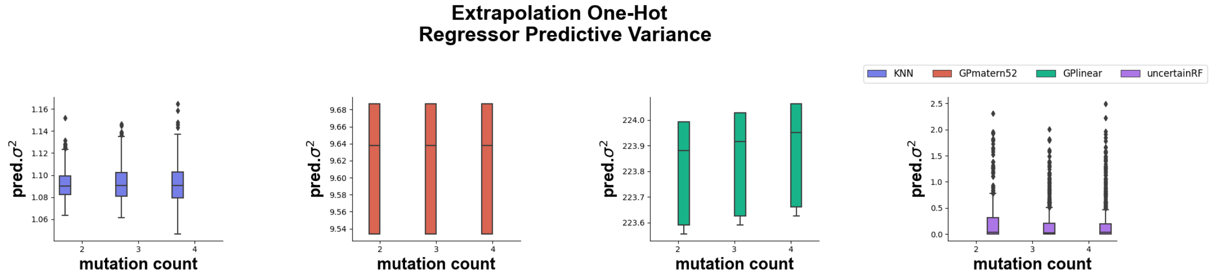

(a) Predictive variances of regressors, data represented via ONE-HOT

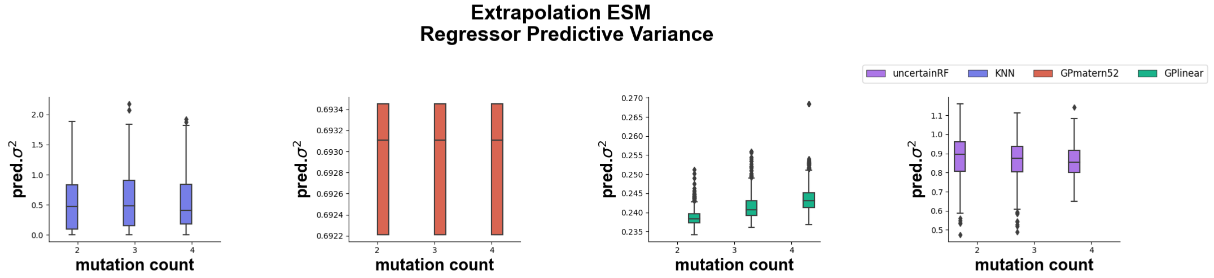

(b) Predictive variances of regressors, data represented via ESM-1B.

Fig U. Predictive variances of regressors tasked with extrapolation of randomly inserted mutations on the β-LACTAMASE dataset.

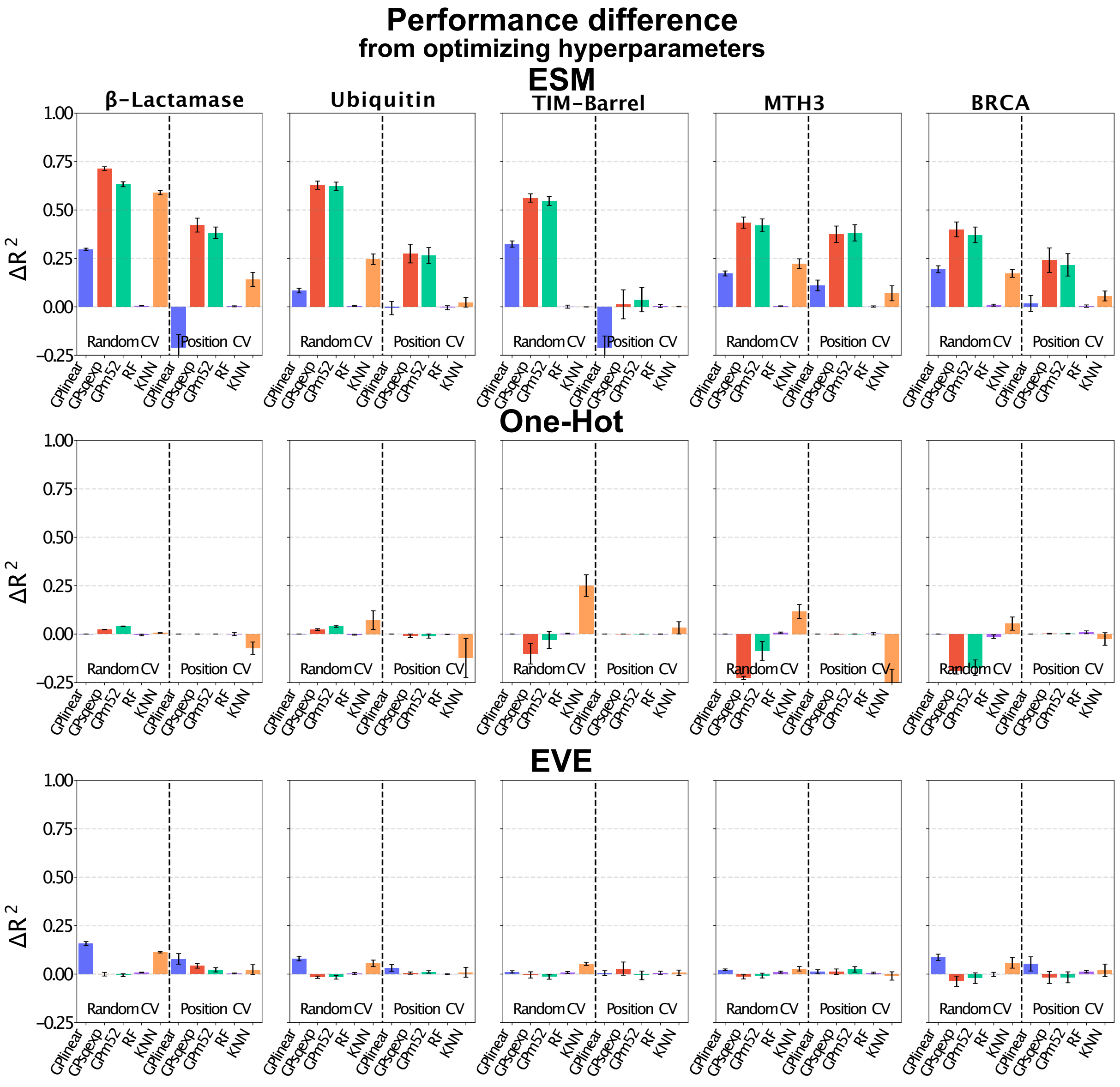

Fig V. Difference in performance (R^2) from parameter optimization for ESM-1B, ONE-HOT, EVE across all regressors (x-axis).

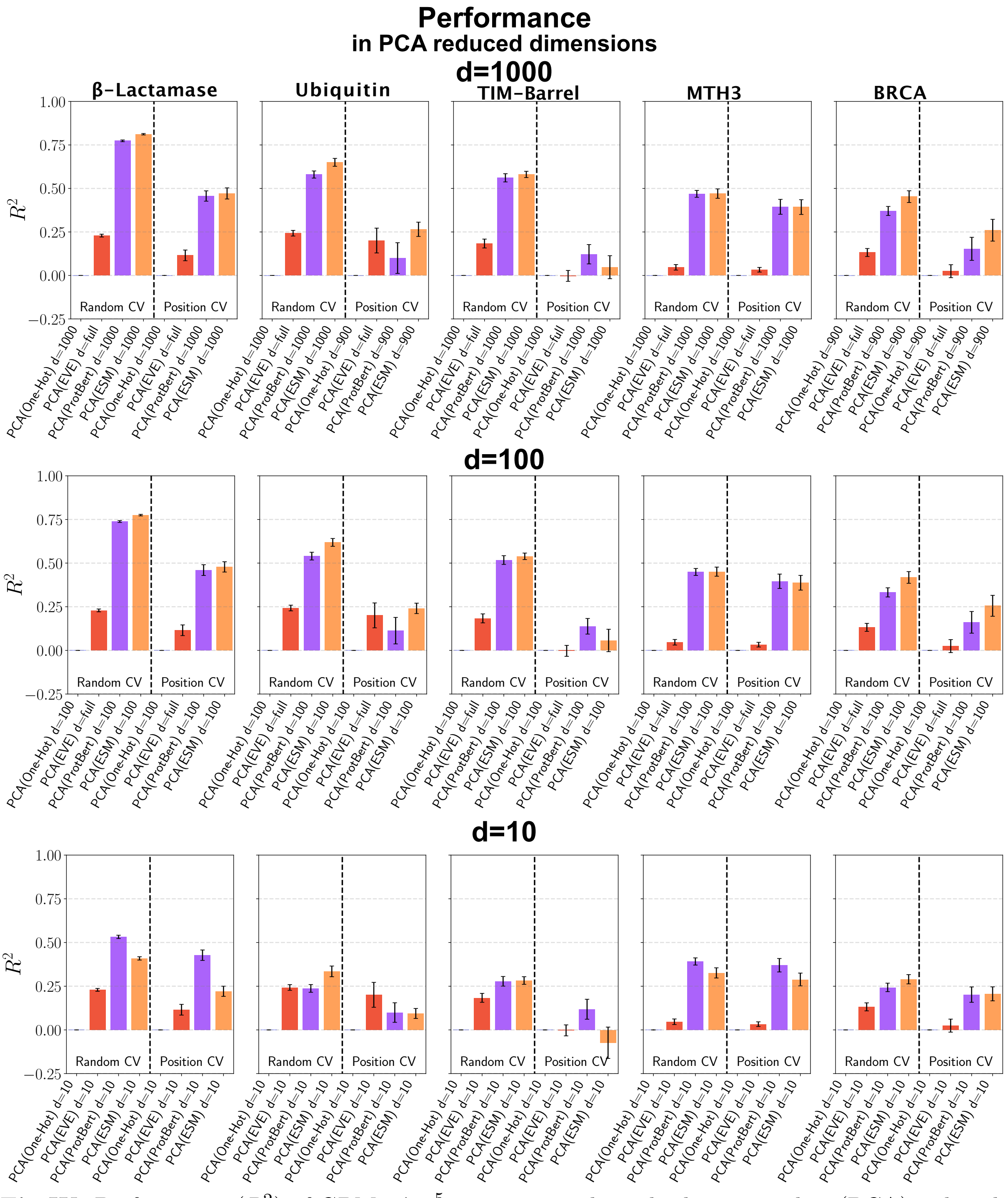

Fig W. Performance (R^2) of GPMatérn$\frac{5}{2}$ regressor on linearly dimensionality (PCA) reduced embeddings for random and positional splitting protocols. Note that for Ubiquitin not all $d = 1000$ representations were stable, hence the dimension has been reduced by an additional $\frac{1}{10}d$ so that $d = 900$, specifically in the positional splits. EVE dimensionality is less than 100, therefore d=10 is the only reduction - d=1000 and d=100 display the full EVE representation.

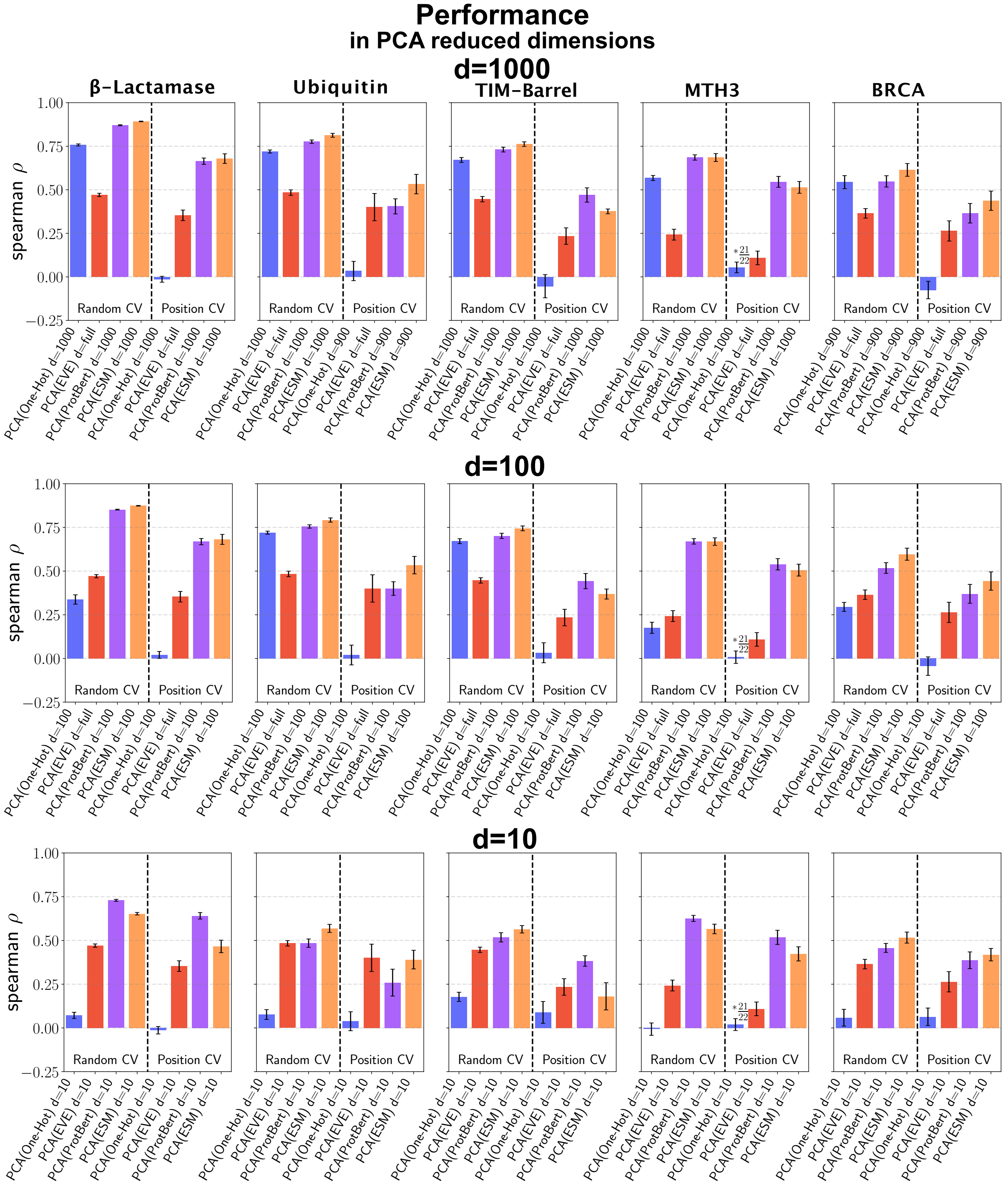

Fig X. Rank correlation (spearman ρ) of GPMatérn$\frac{5}{2}$ regressor on linearly dimensionality (PCA) reduced embeddings for random and positional splitting protocols. Note that for Ubiquitin not all $d = 1000$ representations were stable, hence the dimension has been reduced by an additional $\frac{1}{10}d$, s.t. $d = 900$, specifically in the positional splits. EVE dimensionality is less than 100, therefore d=10 is the only reduction - d=1000 and d=100 display the full EVE representation.

References

[1] Adam J Riesselman, John B Ingraham, and Debora S Marks. Deep generative models of genetic variation capture the effects of mutations. *Nature Methods*, 15(10):816–822, 2018.

[2] Michael A Stiffler, Doeke R Hekstra, and Rama Ranganathan. Evolvability as a function of purifying selection in tem-1 β-lactamase. *Cell*, 160(5):882–892, 2015.

[3] David Mavor, Kyle Barlow, Samuel Thompson, Benjamin A Barad, Alain R Bonny, Clinton L Cario, Garrett Gaskins, Zairan Liu, Laura Deming, Seth D Axen, et al. Determination of ubiquitin fitness landscapes under different chemical stresses in a classroom setting. *Elife*, 5:e15802, 2016.

[4] Jochen Weile, Song Sun, Atina G Cote, Jennifer Knapp, Marta Verby, Joseph C Mellor, Yingzhou Wu, Carles Pons, Cassandra Wong, Natascha van Lieshout, et al. A framework for exhaustively mapping functional missense variants. *Molecular systems biology*, 13(12): 957, 2017.

[5] Yvonne H Chan, Sergey V Venev, Konstantin B Zeldovich, and C Robert Matthews. Correlation of fitness landscapes from three orthologous tim barrels originates from sequence and structure constraints. *Nature communications*, 8(1):1–12, 2017.

[6] Gregory M Findlay, Riza M Daza, Beth Martin, Melissa D Zhang, Anh P Leith, Molly Gasperini, Joseph D Janizek, Xingfan Huang, Lea M Starita, and Jay Shendure. Accurate classification of brca1 variants with saturation genome editing. *Nature*, 562(7726):217–222, 2018.

[7] Liat Rockah-Shmuel, Ágnes Tóth-Petróczy, and Dan S Tawfik. Systematic mapping of protein mutational space by prolonged drift reveals the deleterious effects of seemingly neutral mutations. *PLoS computational biology*, 11(8):e1004421, 2015.

[8] Christopher D Aakre, Julien Herrou, Tuyen N Phung, Barrett S Perchuk, Sean Crosson, and Michael T Laub. Evolving new protein-protein interaction specificity through promiscuous intermediates. *Cell*, 163(3):594–606, 2015.

[9] Jonathan Frazer, Pascal Notin, Mafalda Dias, Aidan Gomez, Joseph K Min, Kelly Brock, Yarin Gal, and Debora S Marks. Disease variant prediction with deep generative models of evolutionary data. *Nature*, 599(7883):91–95, 2021.

[10] Pascal Notin, Mafalda Dias, Jonathan Frazer, Javier Marchena Hurtado, Aidan N Gomez, Debora Marks, and Yarin Gal. Tranception: Protein fitness prediction with autoregressive transformers and inference-time retrieval. In Kamalika Chaudhuri, Stefanie Jegelka, Le Song, Csaba Szepesvari, Gang Niu, and Sivan Sabato, editors, *Proceedings of the 39th International Conference on Machine Learning*, volume 162 of *Proceedings of Machine Learning Research*, pages 16990–17017. PMLR, 17–23 Jul 2022. URL `https://proceedings.mlr.press/v162/notin22a.html`.

[11] Sean R Eddy. A probabilistic model of local sequence alignment that simplifies statistical significance estimation. *PLoS computational biology*, 4(5):e1000069, 2008.

[12] Sean R Eddy. A new generation of homology search tools based on probabilistic inference. In *Genome Informatics 2009: Genome Informatics Series Vol. 23*, pages 205–211. World Scientific, 2009.

[13] Sean R Eddy. Accelerated profile hmm searches. *PLoS computational biology*, 7(10): e1002195, 2011.

[14] Rolf Apweiler, Amos Bairoch, Cathy H. Wu, Winona C. Barker, Brigitte Boeckmann, Serenella Ferro, Elisabeth Gasteiger, Hongzhan Huang, Rodrigo Lopez, Michele Magrane, Maria J. Martin, Darren A. Natale, Claire O'Donovan, Nicole Redaschi, and Lai-Su L. Yeh. Uniprot: the universal protein knowledgebase. *Nucleic Acids Research*, 32:D115–D119, 01 2004. ISSN 0305-1048. doi: 10.1093/nar/gkh131. URL `https://doi.org/10.1093/nar/gkh131`.

[15] Thomas A Hopf, John B Ingraham, Frank J Poelwijk, Charlotta PI Schärfe, Michael Springer, Chris Sander, and Debora S Marks. Mutation effects predicted from sequence co-variation. *Nature biotechnology*, 35(2):128–135, 2017.

[16] Ryan J Tibshirani. Degrees of freedom and model search. *Statistica Sinica*, pages 1265–1296, 2015.

[17] Jonathan Wenger, Nicholas Krämer, Marvin Pförtner, Jonathan Schmidt, Nathanael Bosch, Nina Effenberger, Johannes Zenn, Alexandra Gessner, Toni Karvonen, François-Xavier Briol, et al. Probnum: Probabilistic numerics in python. *arXiv preprint arXiv:2112.02100*, 2021.
