# Supplementary material for: A systematic analysis of regression models for protein engineering
Source: PLoS Comput Biol. 2024 May 3;20(5):e1012061. doi: 10.1371/journal.pcbi.1012061 (PMC11095727; doi:10.1371/journal.pcbi.1012061)
Supplement: Attachment — Submitted filename: Protein_Regression_v3_PlCompBio_point_by_point_response.pdf [file pcbi.1012061.s002.pdf]

We thank all parties involved in the review process for their time and effort. The insightful comments and expressed concerns have been invaluable in enhancing the quality of our work. We have thoroughly reviewed each point raised during the review process and have made significant revisions and additions to the manuscript. In summary, we have:

1. added the representations *ProtT5*, *esm-1v*, *esm-2*, as well as *PSSMs*
2. reassessed performance estimates and calibrations given the additional representations (Figures 2,3 and Supplementary Figures (SF) 1,2,4,5,7,8,9,10)
3. added an ablation study comparing cross validation protocol parameters (SF 17,18)
4. added a new case study to clarify a particular type of bias.
5. made various updates to the text to accommodate the new changes and add to the overall clarity of the paper.
6. Added "protein engineering" to the title to clarify the scope of our study.

See below for a point-by-point response to each of the reviews:

Reviewer #1

> *In this study, the authors investigate different aspects of the application of machine-learning tools to optimization and engineering. Going through the different fundamental steps in a typical supervised machine-learning protocol, they explore challenges and issue related to data, encoding procedures, algorithm selection and performance evaluation. Overall the paper is well-written, the results are presented properly and conclusions drawn are interesting.*

We thank the reviewer for their positive remarks.

> *I only have the following minor concerns:*
>
> *- I would extend the number of mutational datasets explored in this study, to provide a stronger and more general support to conclusions*

We agree with the reviewer that it would have been interesting to extend our study to a broader set of proteins. However, in this work we opted for an in-depth analysis, and the computational demands for the systematic analyses are quite overwhelming. For short sequences using a small embedding (i.e. fitting a GP on an EVE representation with a few samples), the cost are only ∼10-30 seconds (with GPU acceleration). For long sequences on many samples on large embeddings (ie. ESM2 of β-Lactamase) on non GPU-optimized regressors like a Random Forest it takes up to 2 days. Since we in our current approach run all-vs-all comparisons this quickly scales up. For instance, for each dataset one also has to account for: computing the representations (x9 (previously

5)), fitting and optimizing all regressors (x5) for each assessment protocol (x2 main protocols, plus potentially fractional or optimization tasks, which can take multiple days per regressor). Therefore adding one protein ultimately leads to 90 required experiments, which fits multiple models as defined by the protocol, which is 250 (up to 450) additional regressor fits when only assessing Random CV and varying numbers based on sequence length for Position CV. For these reasons, we have opted not to include any new data sets at this time. Instead, in response to the reviews, we have spent our available compute time to expand the experiment to include four new representations (see comments below).

While we agree more proteins would always be better, we do stress that care was taken to select our current datasets to best represent the data available in the literature. In particular, we chose datasets to represent different levels of difficulty, as measured by the performance of unsupervised methods on these systems (now part of ProteinGym (v.0.1) [1] but see also Fig.1 in [2]), ranked from high to low: β-Lactamase, the toxin complex (multiple-variant candidates), BRCA(-BRCT), MTH (DNA function), Ubiquitin, TIM-Barrel, and Calmodulin. These candidates include various lengths of sequences, number of samples, and structural and functional complexity. We therefore believe that the current sequences are meaningful representative cases and sufficient in number to support our main conclusions. However, if there is a particular protein data-set that the reviewer had in mind that would be particularly challenging or useful in revealing additional insights or challenge conclusions drawn in this paper, let us know, and we would be happy to add it to the assessment.

> *- The choice of representative protein representation is in my opinion limited. Regarding evolutionary encoding, I would definitely include in the analysis sequence profiles/PSSMs as directly extracted from multiple-sequence alignments. For what concerns pLMs, the two selected models are in my view a little bit outdated: ESM1-v is probably more well-suited to mutation analysis than ESM1-b, while the most recent EMS2 is now available. Finally, ProtT5 would be more appropriate than ProtBert.*

We agree with the reviewer that the paper would benefit from an updated list of protein language model representations. We have therefore now added ProtT5, ESM-1v, and ESM-2 to our study, and also include a PSSM baseline (see Supplementary section 3.2.1 for details). We find that the updated ProtT5 does indeed yield an improvement over ProtBert, and we've therefore included it in the main figure.
Generally, we do not find a *consistent* significant improvement between the representations ESM-1b, ESM-1v, and ESM-2. Though in many cases ESM-2 yields a better regressor with higher performance on the training split and better parameterized regressors, the test performance is comparable or worse compared to ESM-1b. We have therefore kept ESM-1b in the main part of the manuscript and refer to the supplementary material for all the results on ESM-1v and ESM-2.

We have updated figures 2, 3 and supplementary figures 1, 2, 4, 5, 6, 8, 9, 10, 11 (original numbering) to include the additional representations; SF 1, 2, 4, 5, 7-12 is the

numbering in the revised version.

> *- Also the list of regression algorithms tested could be extended including SVR and/or deep multi-layer perceptrons*

Our problem domain is the low data regime, where predictive uncertainty is instrumental to assess calibration and conduct optimization. We therefore opted to focus on regression methods typically employed in this domain, and consequently did not include MLPs as a model class. To obtain calibrated predictive variance estimates with this particular type of regressor, one would have to consider an ensemble of deep MLP regressors and perhaps conduct MC dropout (or an analogue); computing the expected prediction and predictive variance across the ensemble. We do not disagree with the reviewer that it would be interesting to pursue this avenue, but we highlight again that it would be prohibitively expensive from a computational perspective to do this in the systematic all-vs-all setup of our experiments (see above for details).

Regarding SVR, it was not immediately clear to us how to calculate per-prediction uncertainty estimates within this framework, so we decided to omit this method from our study, also considering that kernel-based methods were already represented through the GP framework. However, if the reviewer has input on uncertainty quantification for SVR and feels strongly about this addition, we would be willing to run the complete round of experiments for this regressor as well.

> *In summary, I believe that the paper could be generally improved just by providing a richer set of case studies, representation techniques and regression algorithms. I believe this would definitely add to the paper, providing a stronger support to conclusions.*

Thank you for the insightful review. We have added four new representations to our study, and believe that this has indeed strengthened the paper.

Reviewer #2

> *The study investigates the impact of various components in protein regression protocols, underlining the significance of data representation and assessment criteria.*
>
> *- correct typos. For instance, in Line 480, the statement "The individual data is published..." should be corrected to "The individual data are published...". Additionally, in Line 483, "The representations are obtained by different means.:" ends erroneously with both a period and a colon. The manuscript frequently employs parenthetical notations that disrupt sentence flow. Utilizing commas might enhance readability, for example: (approximate) in line 28, (variants) in line 62, and (sometimes referred to as wildtype exploration) in line 103.*

We thank the reviewer for pointing out misspellings, inconsistencies, and problems with the flow of our text. We have corrected the manuscript to adopt the suggested changes.

> *- add reference for "e.g. the thermostability of an enzyme" mentioned in line 64.*

Thanks. Added.

> *- In Figure 2 A, while the bottom figure is clear, the upper portion's underlying concept lacks a clear elucidation in the manuscript. It's evident that various datasets and representation methods are being compared, but the meaning of the X and Y axes remains unclear.*

We thank the reviewer for pointing out this source of confusion. We have now adjusted Fig.2A axis labels, as well as row and column titles (MTH3 → T2-MTH, BRCA → BRCA1, ESM → ESM-1b), and figure captions to clarify the values depicted on the axes.

> *- The exploration of distinct CV methods is intriguing. These CV methods are broadly outlined, yet the specifics of their fine-tuning throughout the manuscript remain nebulous. For instance, in Figure 5, is the randomCV reflective of a 10-fold or a 5-fold cross-validation? This detail should be explicitly mentioned when presenting results.*

The specifications of the CV protocol were indeed unclear. We have now updated the figure caption in Figure 5 to reflect this information. Additionally, in the methods section, we now give a full specification of the splitting details under *Protocols*.

> *-An additional point related to the aspect above, in line with the paper's core theme: beyond illustrating discrepancies between CV methods, it would be insightful to demonstrate how certain parameters impact these features, like the effect of K in randomCV or the influence of p in positional CV.*

The impact of k and p for the random and positional CV protocols are indeed an interesting subject for further inquiry. The original manuscript only considered $k = 10$ (standard *Random* CV) and $k = 5$ for fractions of the DMS dataset in the *fractional* CV experiments. To elucidate the question further we have now added a supplementary figure depicting the effect of $k = 2; 3; 5; 7; 15$ and $p = 5; 10; 20; 25$ on the TIM-Barrel dataset and β-Lactamase. We have selected TIM-Barrel since the main results show a striking difference between the random and positional splitting and β-Lactamase because it displays the highest performing regressors and representations. We limit ourselves to these two dataset and three regressors on four representations and the listed number of parameters (k, p) for computational feasibility of the ablation (see discussion about computational constraints to reviewer 1). The results of the effect of CV parameters are

summarized in the new Supplementary Figures 17 and 18, demonstrating the effect for representations: One-Hot, EVE, ProtBert and ESM-1b. We also briefly summarize it as a discussion point in the main text.

> *-A discussion regarding the phenomenon of overfitting and how various choices can influence overfitting assessment would be a valuable addition to the manuscript.*

We thank the reviewer for this suggestion. There are two contexts in which this discussion is potentially important: 1) The choice of a high-capacity regression algorithm, 2) large representations input sizes lead to a large number of parameters in some regression models, which could induce overfitting behavior. We now briefly discuss the problem of overfitting in both these settings (last paragraph in "Protein Representations" and first paragraph in "Regression Algorithms")

References

[1] Pascal Notin, Aaron W Kollasch, Daniel Ritter, Lood Van Niekerk, Steffan Paul, Han Spinner, Nathan J Rollins, Ada Shaw, Rose Orenbuch, Ruben Weitzman, et al. Proteingym: Large-scale benchmarks for protein fitness prediction and design. In *Thirty-seventh Conference on Neural Information Processing Systems Datasets and Benchmarks Track*, 2023.

[2] Adam J Riesselman, John B Ingraham, and Debora S Marks. Deep generative models of genetic variation capture the effects of mutations. *Nature methods*, 15(10):816–822, 2018.
